# Temperature-Dependent Ferroelectric Properties and Aging Behavior of Freeze-Cast Bismuth Ferrite–Barium Titanate Ceramics

**DOI:** 10.1021/acsami.4c03002

**Published:** 2024-04-05

**Authors:** Bastola Narayan, Zihe Li, Bing Wang, Astri Bjørnetun Haugen, David Hall, Hamideh Khanbareh, James Roscow

**Affiliations:** 1 Department of Mechanical Engineering, University of Bath, Bath BA2 7AY, U.K.; 2 Department of Materials, University of Manchester, Manchester M13 9PL, U.K.; 3 Department of Energy Conversion and Storage, Technical University of Denmark, Copenhagen 2800, Denmark

**Keywords:** ferroelectrics, lead-free, high temperature, porous piezoelectric, piezoelectric sensors, energy harvesting

## Abstract

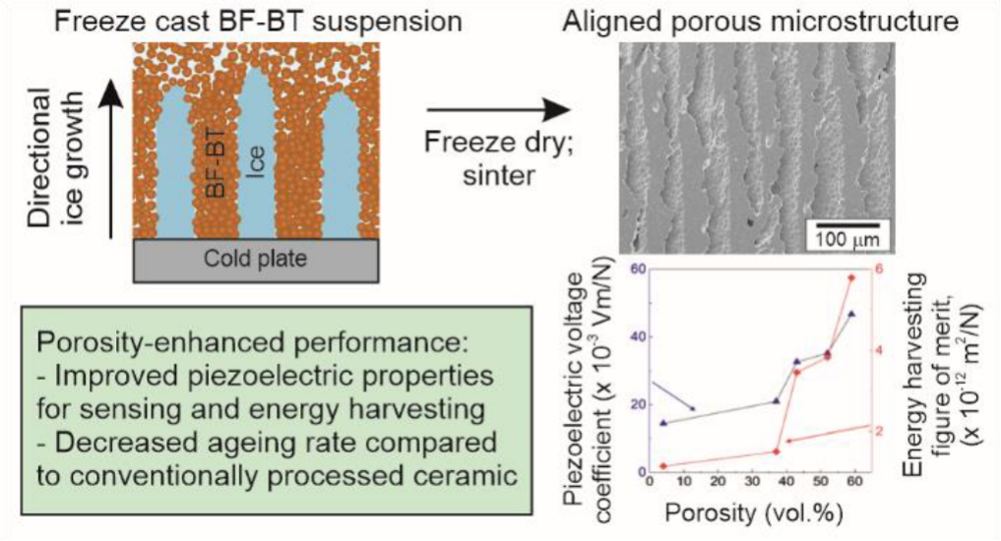

Lead-free BiFeO_3_–BaTiO_3_ (BF-BT) piezoceramics have sparked considerable interest in recent years due to their high piezoelectric performance and high Curie temperature. In this paper, we show how the addition of highly aligned porosity (between 40 and 60 vol %) improves the piezoelectric performance, sensing, and energy harvesting figures of merit in freeze-cast 0.70BiFeO_3_–0.30BaTiO_3_ piezoceramics compared to conventionally processed, nominally dense samples of the same composition. The dense and porous BF-BT ceramics had similar longitudinal piezoelectric coefficients (*d*_33_) immediately after poling, yet the dense samples were observed to age faster than those of porous ceramics. After 24 h, for example, the porous samples had significantly higher *d*_33_ values ranging from 112 to 124 pC/N, compared to 85 pC/N for the dense samples. Porous samples exhibited 3 and 5 times higher longitudinal piezoelectric voltage coefficient *g*_33_ and energy harvesting figure of merit *d*_33_*g*_33_ than dense samples due to the unexpected increase in *d*_33_ and decrease in relative permittivity with porosity. Spontaneous polarization (*P*_s_) and remnant polarization (*P*_r_) decrease as the porosity content increased from 37 to 59 vol %, as expected due to the lower volume of active material; however, normalized polarization values with respect to porosity level showed a slight increase in the porous materials relative to the dense BF-BT. Furthermore, the porous ceramics showed improved temperature-dependent strain–field response compared to the dense. As a result, these porous materials show excellent potential for use in high temperature sensing and harvesting applications.

## 1 Introduction

There is a growing requirement to develop sensor materials that can operate under extreme environments for demanding applications within, for example, the aerospace, oil and gas, and automotive industries.^1,2^ Continuous monitoring of engineering systems operating under a combination of high temperature and high mechanical loads, and often within highly corrosive environments, is critical for predicting and ideally extending the lifetime and reliability of components while reducing maintenance costs. This is an important step in avoiding component failure that can pose significant risks to the environment as well as ecological and/or human health. Piezoelectric transducers are critical for health monitoring and nondestructive sensing^3^ as they can both transmit and detect ultrasonic waves, which can be used to detect faults and monitor flow and corrosion in engineering systems. The ability of these materials to scavenge ambient energy from harsh environments to directly power sensor networks has also gained interest in recent years.^4^

The piezoelectric properties of interest for sensing applications include piezoelectric charge coefficients, *d*_*ij*_, dielectric permittivity at constant stress, ε_33_^*T*^, the piezoelectric voltage coefficient,
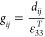
1and the electromechanical coupling coefficient,
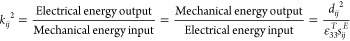
2where ε_*ij*_^*E*^ is the elastic compliance under constant electric field. The subscripts *i* and *j* refer to the direction of polarization and mechanical response, respectively. For energy harvesting, the figure of merit for a stress-driven system is given by^5^
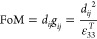
3

Engineering materials with high piezoelectric charge coefficient and low permittivity are favorable for maximizing both the sensing and energy harvesting performance of a piezoelectric device. In polycrystalline piezoceramics, however, this is challenging as *d*_*ij*_ and ε_33_^*T*^ are intrinsically linked.^6^ An effective method of decoupling these two properties is forming composites from a piezoelectric material with a piezopassive, low permittivity second phase.^7^ Traditional piezocomposites manufactured for hydrophones or medical ultrasound transducers tend to use polymer second phases; however, these are unsuitable in high temperature applications. Porous piezoceramics, where the porosity is used as an active component in controlling the properties, avoid the temperature-stability issues of polymers while providing a route to increasing in relevant figures of merit.

Porous piezoceramics have been demonstrated to have excellent properties for sensing and harvesting applications, with significant improvements in voltage sensitivity^7^ compared to conventionally processed polycrystalline ceramics, for example, *g*_33_ > 400 × 10^–3^ V m/N was reported for porous lead zirconate titanate (PZT)^8^ compared with <50 × 10^–3^ V m/N for dense PZT.^9^ High volumes of porosity also decrease the acoustic impedance of the active material enabling improved mechanical energy transfer between active and passive components, thereby providing a further benefit for sensing.^10^ Tailoring porous microstructures to provide a high degree of pore and ceramic phase alignment has been shown to be very effective for optimizing their piezoelectric and dielectric properties. The highest *d*_33_ coefficients occur when pore channels are aligned parallel to the poling direction, and the lowest permittivity is observed when anisometric pores are oriented perpendicular to the poling direction.^8,11,12^ Aligning microstructures to the poling direction reduces local field inhomogeneity and increases poling efficiency,^13^ as well as causing uncertainty in accurately evaluating the measured ferroelectric properties.^12^ It has been shown recently that the porosity can alter both the intrinsic (lattice) and extrinsic (domain wall) contributions to the piezoelectric and dielectric responses of ferroelectric ceramics.^14^ A relaxation in the residual stress and intergranular clamping induced by poling was observed in porous barium titanate with 52 vol % porosity fabricated via the freeze casting method. This led to an increase in domain wall mobility under an electric field compared to conventionally processed, low porosity ceramic. Residual stress states in these materials have also been linked to structural changes, with the ratio of tetragonal and orthorhombic polymorphs observed to change with porosity in barium titanate modeled using the finite element method.^15^ This recent research indicates that the presence of porosity can be used to tailor the effective properties of piezoceramics in terms of figures of merit but also their physical properties at the lattice and domain scale.

Porosity can be used to tune the electromechanical properties of piezoelectric ceramics, but absolute piezoelectric charge coefficients, permittivity, and temperature stability are all dominated by the composition. For sensing applications at high temperatures, they are limited by their Curie point, which is the temperature at which ferroelectrics lose their spontaneous polarization due to a phase transition. The most widely used piezoelectric ceramic in commercial devices is lead zirconate titanate (PZT), which can be compositionally engineered to have a high Curie point of up to 360 °C with longitudinal piezoelectric charge coefficients of *d*_33_ ∼ 450 pC/N for PZT-5A.^16^ However, regulations targeted at reducing the use of toxic lead in electrical components has led to an expansion of research into lead-free piezoceramics.^17^ Of the lead-free piezoceramics of interest for high temperature applications, bismuth ferrite–barium titanate (BF-BT) ferroelectrics have shown great promise, with Curie points of over 400 °C and piezoelectric charge coefficients of >200 pC/N.^18^

Various strategies have been used to promote high piezoelectric properties in BF-BT ceramics, including doping^19^ and quenching^20^ to lock in desirable microstructures and defect structures. In applications involving high temperature actuators, the electrostrain should remain steady even as the temperature is elevated. It has been demonstrated that BF-BT based compositions have an exceptional thermal stability of *d*_33_ with a high depolarization temperature (*T*_d_) above 500 °C.^21^ The *K*^+^-modified 0.63BF-0.37BT ceramic at 100 °C exhibited excellent electromechanical properties, with an effective *d*_33_^*^ of 861 pm/V at an electric field of 30 kV/cm.^22^ A stable electrostrain about 0.3% under relatively high field amplitude of 60 kV/cm at 175 °C has been reported for the 0.67BF-0.33BT modified with a relaxor ferroelectric Sr_0.8_Bi_0.1_□_0.1_ TiO_2.95_^23^ (where □ refers to the A-site vacancies). On the other hand, Bi(Mg_2/3_Nb_1/3_)O_3_-modified BF-BT-based multilayer actuators were found to exhibit twice the strain displacement at 150 °C compared to that at room temperature at 7 kV/mm (1.5 and 3 μm, respectively).^24^ Similarly, Nd-doped 0.70BiFeO_3_–0.3BaTiO_3_ ceramics exhibit an increase in unipolar electrostrain from 0.15% at RT to 0.4% at 150 °C at an applied field amplitude of 60 kV/cm, as well as a doubling of the *d*_33_^*^ value at 150 °C relative to RT.^25^ Even though BF-BT is a promising lead-free candidate for high temperature applications, only a few studies of temperature dependent dielectric and ferroelectric properties have been reported.^21−23,26^ Furthermore, there have been no temperature-dependent studies on the ferroelectric properties of porous BF-BT conducted to date. These are required to understand their electromechanical properties over a wide temperature range to evaluate their potential as high temperature transducers.

High temperature piezoelectric properties of porous ferroelectric ceramics have not been widely reported in general, although temperature-dependent dielectric properties have been studied. The dielectric behavior aligns with that seen in dense ferroelectric ceramics albeit with slight perturbations in the Curie point. For example, an increase in the Curie point was reported in porous barium zirconium calcium titanate (BCZT)^27,28^ and PZT ceramics,^29^ whereas a decrease was reported for porous 0.36BiScO_3_–0.64PbTiO_3_ and barium calcium titanate. In some cases, no significant change in Curie point was observed as a function of pore fraction, e.g., in porous barium strontium titanate^30^ or barium zirconium titanate.^31^ The exact mechanism for any slight change in Curie point with increasing porosity is currently unconfirmed although Zhang et al.^29^ proposed it was related to a stress relaxation around the pores.

While the effect of porosity on phase transition temperatures is of interest from a scientific perspective, the significance of slight changes in Curie point for a given composition is relatively limited for practical applications. More intriguing is the effect of the porosity on the piezoelectric properties. So far, Khansur et al.^32^ have provided the only in-depth study to date, recently showing that the piezoelectric properties of porous PZT, formed using the burned-out polymer spheres (BURPS) method with cellulose as the pore former, were maintained to around 350 °C. The highest porosity sample with 50 vol % porosity showed the least sensitivity to increases in temperature, with only a 4% variation in the *d*_33_ up until the depolarization temperature, *T*_d_. Due to the increase in permittivity with temperature, the voltage sensitivity, *g*_33_, decreased as a function of temperature. The Curie point was relatively insensitive to porosity, with a very small increase of 2 °C observed in the 40 and 50 vol % porosity samples. These results indicate that porous PZT can be used for high temperature sensing, provided that a composition with a suitable *T*_C_ and *T*_d_ is used. As BF-BT is the most promising lead-free alternative for high temperature applications, it is of interest to study its temperature-dependent piezoelectric, ferroelectric, and dielectric properties.

In this study, freeze cast BF-BT was investigated to uncover the effect of porosity on its electromechanical properties. Conventionally processed “dense” and porous 0.70BiFeO_3_ + 0.30 BaTiO_3_ + 1 mol % MnO_2_ (BFBT30) samples were prepared using the solid-state method and freeze casting techniques, respectively. Freeze cast samples with aligned porous microstructures and a range of porosities were fabricated. Their room temperature piezoelectric properties and temperature-dependent ferroelectric properties were measured to evaluate their sensing and energy harvesting figures of merit and the effect of porosity on polarization–electric field and strain–field behavior. X-ray photoelectron spectroscopy (XPS) was used to investigate changes in the defect concentration driven by bismuth loss during sintering. Rayleigh analysis was performed to evaluate the intrinsic and extrinsic contributions to dielectric properties for dense and porous BF-BT. Piezoelectric charge coefficients were monitored as a function of time after poling to provide insight into aging behavior in these materials.

## 2 Methods

### 2.1 Sample Preparation

The conventional solid-state route was used to prepare solid solutions of 0.70BiFeO_3_ + 0.30 BaTiO_3_ + 1 mol % MnO_2_ (BFBT30) ceramics, with manganese being added to reduce dielectric loss and improve electrical resistance. Analytical grade high purity raw ceramic oxide powders (Bi_2_O_3_ (99%. Alfa Aesar), Fe_2_O_3_ (99%, Honeywell), BaCO_3_ (99%, Alfa Aesar), TiO_2_ (99%, Sigma-Aldrich)) were milled thoroughly according to stoichiometric proportions using a vibration milling machine (Pilamec MEGAPOT) for 24 h with propan-2-ol and yttrium-stabilized zirconia balls as the milling media. The slurry was dried overnight under an infrared lamp and calcinated at 830 °C for 3 h. Calcined samples were then milled for 24 h to break particle agglomerations and dried to yield fine calcined powder.

**Figure 1:**
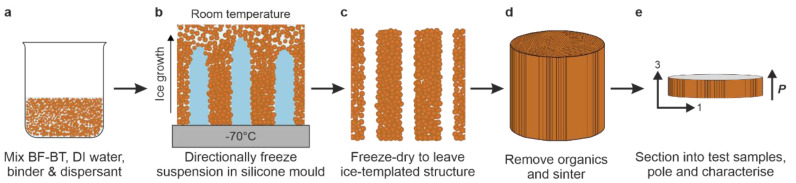
Schematic showing the freeze casting process of the BF-BT. (a) Suspension preparation, (b) freeze casting of suspension, (c) freeze-drying and orientation of the lamellae, (d) sintered porous BFBT30, and (e) sectioning of the samples to the perpendicular to freezing direction.

Porous BFBT30 ceramics were prepared using the freeze casting technique; a schematic of the process is presented in Figure 1. Slurries of different solid loadings (SL = 20, 25, 30, and 35 vol %) of BFBT30 calcined powders in deionized water with 1 wt % polyethylene glycol (*m*_w_ = 8000 g/mol, Sigma-Aldrich, U.K.) as a binder and 1 wt % ammonium polyacrylate (HydroDisper A160, Shenzhen Highrun Chemical Industry Co. Ltd., China) as a dispersant were mixed on a rotary ball mill for 24 h; both weights of the organic additives were calculated relative to the ceramic mass. The slurries were cast in open polydimethylsiloxane (PDMS) cylindrical molds (height ∼20 mm, diameter ∼12 mm), secured on the bottom side with aluminum adhesive tape and placed on a precooled aluminum plate at −70 °C with an ultralow temperature circulator (Lauda RP2090, U.K.). Frozen bodies were freeze-dried to remove ice for 24 h (Mini Lyotrap, LTE Scientific, U.K.). The green freeze-dried bodies were then transferred to a closed alumina crucible for sintering at 1010 °C for 4 h with 3 °C per minute heating and cooling rate and a dwell stage at 500 °C for 2 h to remove organic additives in a Lenton muffle furnace. Sintered samples were mounted in wax and cut into thin pellets of 1.5–2.0 mm thickness perpendicular to the freezing direction, see Figure 1e, using a diamond wire saw (STX-202A, MTI Corp., USA). Individual pellets were cleaned in acetone and dried in an oven at 120 °C for 1 h. Silver paste (RS 186–3600, RS Components) was applied to form conductive electrodes on surfaces of the pellets and cured at 120 °C for 2 h in an oven prior to electrical characterization. For comparison, dense pellets of the same chemical composition were prepared by mixing 2.5 wt % of polyethylene glycol as a binder with calcined powder, pressed uniaxially into pellets in a 13 mm die, and sintered at identical conditions to the porous ceramics. Sintered pellets were then polished on either side before applying the silver paste and cured at 120 °C for 2 h for the electrical measurements.

### 2.2 Materials Characterization

The relative density was calculated from the mass and geometry of the samples and compared to the theoretical density:
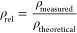
4A theoretical density of 7.56 g/cm^3^ was used, which was calculated from the refined lattice parameters obtained from laboratory X-ray diffraction (XRD) data. The bulk porosity volume was estimated from the relative density using
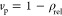
5Microstructures were investigated using scanning electron microscopy (SEM, Hitachi SU-3900, Japan) with samples mounted in resin for polishing (EcoMet 250Pro, Buehler, U.K.). Laboratory XRD patterns were collected in transmission mode from crushed powders and sintered specimens. Powders were annealed at 600 °C for 1 h to remove mechanical stresses induced during crushing. High energy XRD patterns were collected on beamline I15 at the Diamond Light Source. A monochromatic photon source with an energy of 78.395 eV was used, and diffraction patterns were collected in transmission with a PerkinElmer flat panel detector. XRD patterns were collected from a dense and a porous (solid loading, SL = 30 vol %, and pore volume, *v*_p_ + 43 vol %) samples before poling, immediately after contact poling at 5 kV/mm for 5 min at room temperature in silicone oil, and at various times up to 21 h after poling to inform on any structural relaxations that took place. X-ray photoelectron spectroscopy (XPS) was conducted using a Thermo NEXSA XPS fitted with a monochromated Al Kα X-ray source (1486.7 eV), a spherical sector analyzer, and three multichannel resistive plate and 128 channel delay line detectors. All data were acquired at 19.2 W with an X-ray beam size of 400 μm × 200 μm. Survey scans were captured at 200 eV, whereas high-resolution scans were recorded at 40 eV. Electronic charge neutralization was accomplished with a dual-beam low-energy electron/ion source (Thermo Scientific FG-03). The ion gun current was 150 μA and the voltage was 45 V. All sample data were acquired at a pressure less than 10^–8^ Torr and a temperature of 294 K. The data were examined using CasaXPS v2.3.20 rev1.0.

Room temperature dielectric measurements were conducted using impedance spectroscopy (Solartron SI 1260 and 1296 Dielectric Interface, U.K.). Pellets were corona poled with a 16 kV point source at 37.5 mm distance from samples for 30 min and rested for 4 h before making an initial longitudinal piezoelectric charge coefficient (*d*_33_) measurement on poled samples using a PiezoMeter PM300 (Piezotest, Singapore). Corona poling was used as it is effective for poling porous piezoceramic samples while avoiding dielectric breakdown;^33^ dense samples were poled under the same conditions to enable a direct comparison. The poling voltage was selected based on preliminary tests where it was found to yield the highest *d*_33_ values without damaging the samples. Room temperature polarization-electric field (*P*–*E*) loops were measured with a Precision Premier II Loop Tracer (Radiant Technologies, USA). Temperature dependent *P*–*E* and strain–field (*S*–*E*) hysteresis measurements were conducted using a piezo-/ferroelectric tester (TF1000, aixACCT GmbH) at 1 Hz over the temperature range from 25 to 200 °C on samples infiltrated with silicone oil (200 AK, Wacker, Germany). *P*–*E* hysteresis loops were measured at subcoercive field levels for dense and a porous sample (SL = 30 vol %) at room temperature as well as at elevated temperature (100 °C) to quantify the intrinsic and extrinsic contributions to the dielectric permittivity using Rayleigh analysis. The relative permittivity in the Rayleigh regime can be expressed as
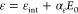
6where *ε*_int_ is reversible (intrinsic), *α*_ε_ is the Rayleigh coefficient responsible for the irreversible (extrinsic) contribution, and *E*_0_ is the electric field amplitude in the subcoercive region. The permittivity in eq 6 was calculated from polarization–field loops such that
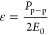
7where *P*_p__–p_ is the peak-to-peak polarization at applied field amplitude *E*_0_. The validity of the Rayleigh analysis depends on the linearity of the ε(*E*_0_) relationship in the measured field range. From eq 6, the Rayleigh coefficient, *α*_ε_, is calculated from the gradient while the initial permittivity, ε_int_, is determined from the zero-field intercept.

## 3 Results and Discussion

### 3.1 Structural Characterization

**Figure 2:**
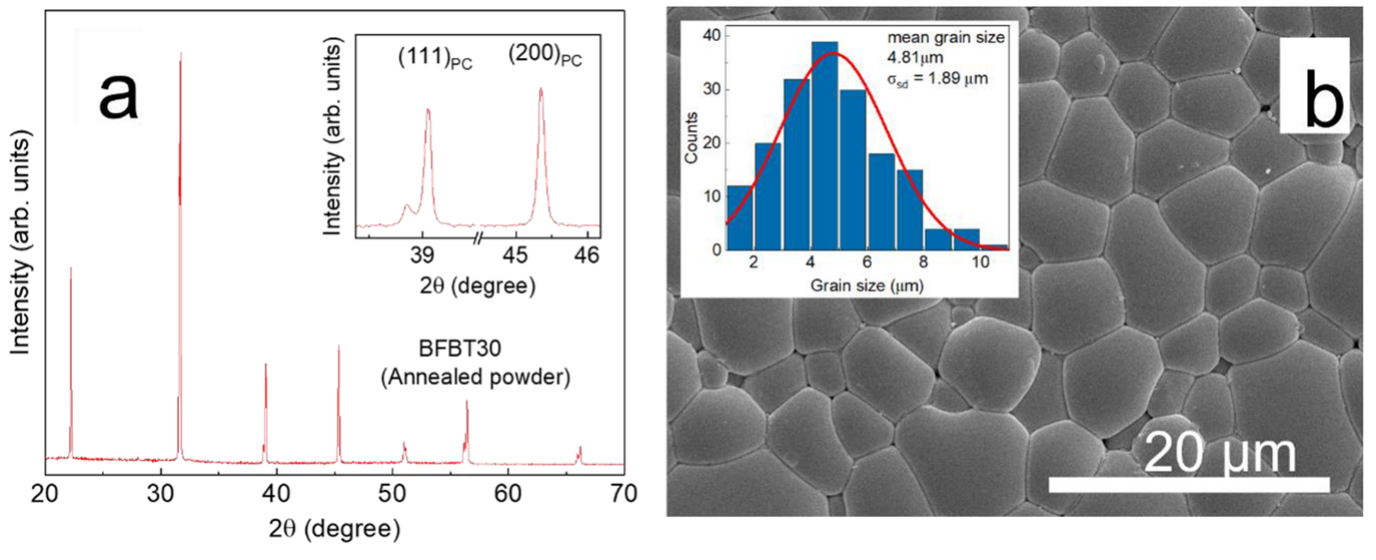
(a) XRD pattern of sintered BFBT30 powder sample after annealing at 600 °C for 1 h (inset shows the magnified view of (111) and (200) peak profiles and (b) SEM micrograph of the dense BFBT30 sintered surface with grain size analysis in the inset.

The porosity, *v*_p_, of the dense reference sample was 4 vol % for the dense reference sample (i.e., relative density *ρ*_rel_ = 0.96), and average pore volumes of *v*_p_ = 37 vol %, 43 vol %, 52 vol %, and 59 vol % were calculated for samples freeze cast from suspensions with solid loading, SL = 35 vol %, 30 vol %, 25 vol %, and 20 vol %, respectively. The laboratory XRD pattern of the dense BFBT30 ceramic showed a pure perovskite phase with no visible impurities; see Figure 2a. The distinct splitting of 111 Bragg peaks and singlet 200 Bragg peaks confirmed the rhombohedral (*R*3*c*) symmetry, which is consistent with previously reported structure from the same composition.^19^ There was no significant change in the structure observed between the XRD of the porous shown in Figure S1 compared to the dense BF-BT. An SEM micrograph of a dense BFBT30 sintered surface is shown in Figure 2b, with grain diameters ranging from 4 to 6 μm. The average diameter of the grains was measured from a polished surface (Figure S2) using ImageJ software, and a histogram was fitted using the Gaussian function, yielding a mean grain size of 4.81 μm and standard deviation σ_sd_ 1.89 μm.

**Figure 3:**
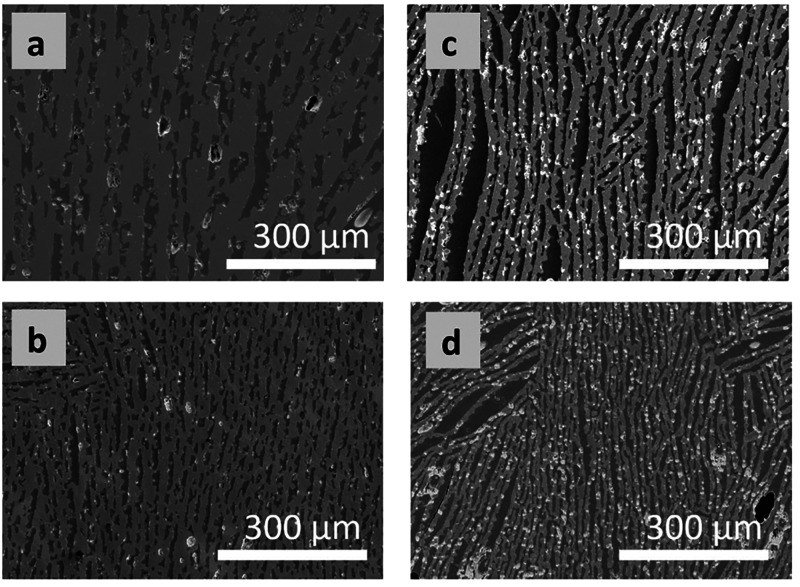
SEM micrographs of the freeze cast BFBT30 with (a) pore volume *v*_p_ = 37 vol %, (b) *v*_p_ = 43 vol %, (c) *v*_p_ = 52 vol %, and (d) *v*_p_ = 59 vol %. Freezing direction is perpendicular to the plane of the images.

The microstructural features of the porous BFBT30 samples with a freezing direction perpendicular to the image plane are displayed in Figure 3a–d. The bright patches in the micrograph represent the charging effects on artifacts left behind from polishing the ceramic/epoxy composite. The porosity of the freeze cast bodies increased slightly from the bottom (surface in contact with freezing surface) to the top of the scaffold. Bottom to top, the range of porosities of the porous pellets were 35–39 vol %, 40–45 vol %, 48–54 vol %, and 56–63 vol % for SL = 35 vol %, SL = 30 vol %, SL = 25 vol %, and SL = 20 vol %, respectively. Micrographs of all porous samples with the freezing direction parallel to the image plane are shown in Figure S3 at two different magnifications. In general, good microstructural alignment, i.e., anisometric pores separated by dense ceramic channels oriented to the freezing direction, were observed in the porous BF-BT samples. The lamellae width was larger (∼38 μm) and less well-ordered in the freezing direction in the 35 vol % solid loading samples because the interconnecting layers between the lamellae were densely packed due to the increased viscosity of the suspension with higher solid loading that inhibits the ice-templating process. The pore alignment relative to the freezing direction was also less consistent in the SL = 35 vol % ceramics, and even when regions of high pore alignment were observed in the SEMs, pores within ceramic lamellae at ∼45° angles were present (Figure S3a,b), which can significantly reduce local field strength in the ferroelectric phase during poling and electromechanical characterization.^34^ The lamellae thickness decreased with solid loading, whereas alignment of the BF-BT and pore channels increased. The free surface area per unit volume, i.e., the amount of ceramic at a pore surface, in porous BFBT30 ceramics therefore also increased as solid loading decreased. Figure S4 shows the SEM micrographs of polished surface of porous BFBT30 with pore volumes of 37 vol %, 43 vol %, 52 vol %, and 59 vol %, respectively, in the suspension with plane of image parallel to the freezing direction. The average grain sizes of the porous specimens ranged from 7 to 9 μm, shown by histograms plotted alongside the micrographs in Figure S4, which was larger than the average grain size of the dense samples (4.81 μm).

**Figure 4:**
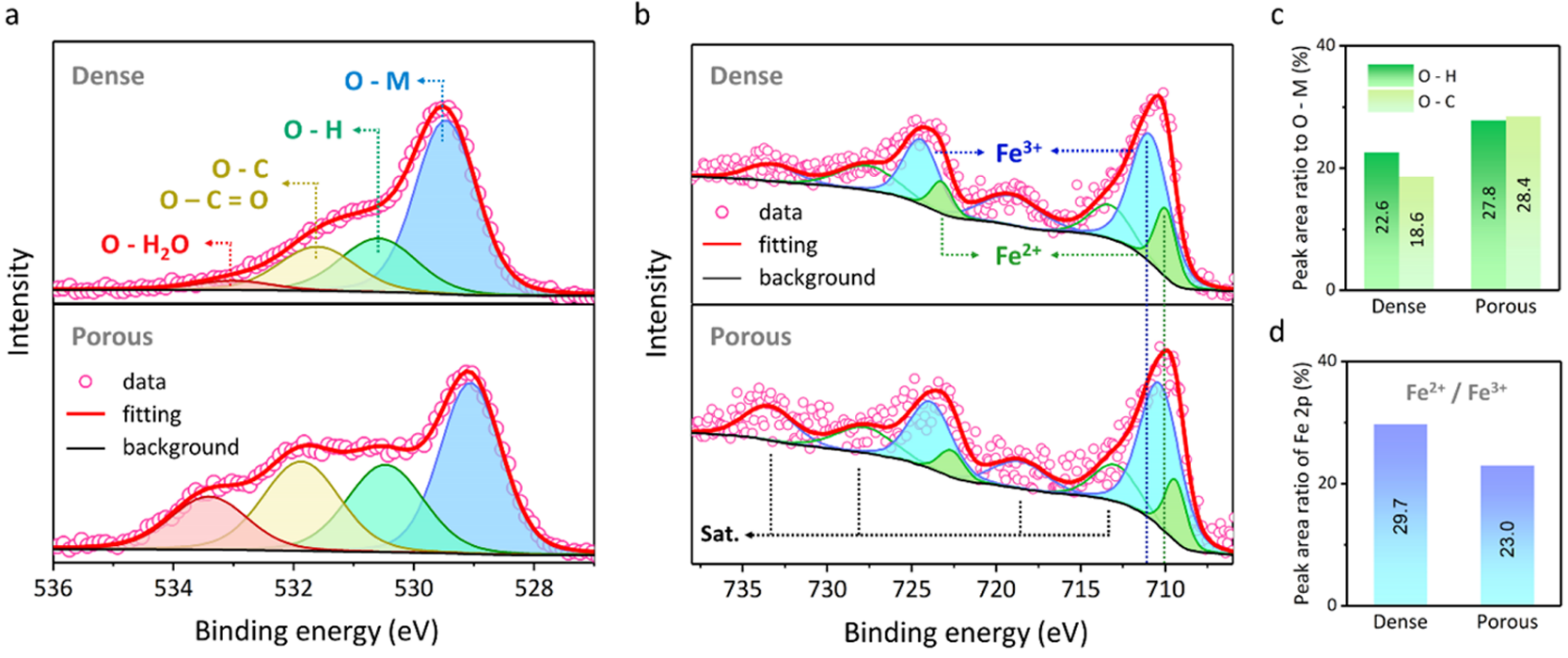
X-ray photoelectron spectra (XPS) of dense and porous (*v*_p_ = 43 vol %) BFBT30 ceramics. Narrow scans of (a) O 1s and (b) Fe 2p peaks, and the peak area ratio analysis of (c) O 1s and (d) Fe 2p orbits for dense and porous BF-BT.

X-ray photoelectron spectroscopy (XPS) was used to investigate local structural differences between the dense and the porous BF-BT. Narrow scans of the XPS spectra of the O 1s and Fe 2p orbits are shown in Figure 4a and Figure 4b, respectively, for dense and porous (*v*_p_ = 43 vol %) BF-BT. The data were first calibrated with the C 1s peak of adventitious carbon^35^ shown in Figure S5, before the peaks were assigned using a Gaussian/Lorentzian function. In the XPS spectra of O 1s, the curve was fitted into four components^36^ where (i) the blue peak was assigned to the bond between oxygen atom and metallic atom in the lattice (O–M), (ii) the green peak was due to the bond between oxygen atom and hydrogen atom (O–H), (iii) the yellow peak was due to the bond between oxygen atom and carbon atom (O–C/O–C=O), and (iv) the red peak was related to the bond between oxygen atom and the adsorbed water molecules (O–H_2_O). An increase in the peak area of the O–H, O–C/O–C=O, and O–H_2_O peaks was observed in the porous BF-BT. In some works^37,38^ the O 1s peaks at ∼531 eV have been correlated to the presence of oxygen vacancy in the ceramics lattice since the vacancy can promote the adsorption of small molecules, e.g., OH and CO_2_,^39^ leading to an increase in the peak intensity at ∼531 eV. Therefore, an analysis of the peak area ratios of O–H to O–M and O–C to O–M was performed in Figure 4c. These two ratios were both higher for the porous sample than for the dense one, indicating that more oxygen vacancies were present in the porous BF-BT.

To further understand the effect of porosity on the defect structure of BF-BT, the XPS spectra of Fe 2p was considered, see Figure 4b. The curve was fitted into eight components including (i) two spin–orbit splitting peaks of the Fe^2+^ 2p (in green), (ii) two spin–orbit splitting peaks of the Fe^3+^ 2p (in blue), and (iii) four satellite peaks of the above four peaks, following the corresponding peak with a slightly higher binding energy. Compared to the dense BF-BT ceramic, both the Fe^3+^ 2p and Fe^2+^ 2p peaks of the porous sample exhibited a peak shift toward a lower binding energy of ∼0.6 eV. A similar peak shift was also observed in the XPS spectra of Ba 3d and Bi 4f, shown in Figures 4c and S5, with a decrease in the binding energy of ∼0.4 eV in the porous BF-BT. A lower binding energy indicates a higher electron density around the atoms,^40^ which can arise due to the higher concentration of oxygen vacancies in the lattice that acts to pull more electrons toward the metal atoms and lower the observed binding energy. The valence change of Fe ions in the BF-BT ceramics was studied through a peak area analysis, shown in Figure 4d. The lower peak area ratio of Fe^2+^ 2p to Fe^3+^ 2p of the porous BF-BT indicated that fewer Fe^3+^ ions were reduced to Fe^2+^ ions than in the dense material.

Based on the XPS spectra, the effect of the introduced porosity on the defect chemistry in the BF-BT ceramics is now discussed. As one of the main lattice defects in BF-BT ceramics, oxygen vacancies (V_O_^••^) can be formed due to the bismuth loss by evaporation (eq 8) and/or the reduction of Fe^3+^ ions (Fe_Fe_^×^) to Fe^2+^ ions (Fe_Fe_^*’*^) (eq 9):^41^
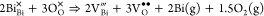
8
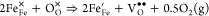
9

The data suggests that the higher oxygen vacancy concentration in the porous BF-BT arises due to the bismuth loss described by eq 8, as the peak area ratio of Fe^2+^ to Fe^3+^ was lower in the porous BF-BT than in the dense BF-BT (Figure 4d), which indicates that the influence of porosity on Fe^3+^ reduction was minor. It has also been claimed that the formed V_O_^••^ in BF-BT can be further consumed by the oxidation of Fe^3+^ ions (Fe_Fe_^×^) to Fe^4+^ ions (Fe_Fe_^•^)^41^ as described below:
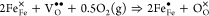
10

However, in this work no significant peak was observed at the binding energy of ∼736 eV that relates to the expected position of the 2p orbit of Fe^4+^ (Fe_Fe_^•^) (Figure 4b)^42^ and, as such, the mechanism described by eq 10 on both the dense and porous BF-BT does not appear to be significant here. The observed increased oxygen vacancy concentration is therefore most likely related to increased bismuth loss in the porous BF-BT during sintering, which is promoted by the higher surface area due to the presence of porosity. Skin effects have been observed previously at the surfaces of dense bismuth-based ferroelectric ceramics, whereby there is a strain-induced change in the composition and lattice parameters within ∼20 μm of the surface that decays into the bulk.^43^ These effects were not observed in the porous materials here in either XRD or XPS, which may be related to the scale of the microstructural features in the porous BF-BT with ceramic channels typically less than 30 μm in width.

### 3.2 Room Temperature Piezoelectric and Dielectric Properties

**Figure 5:**
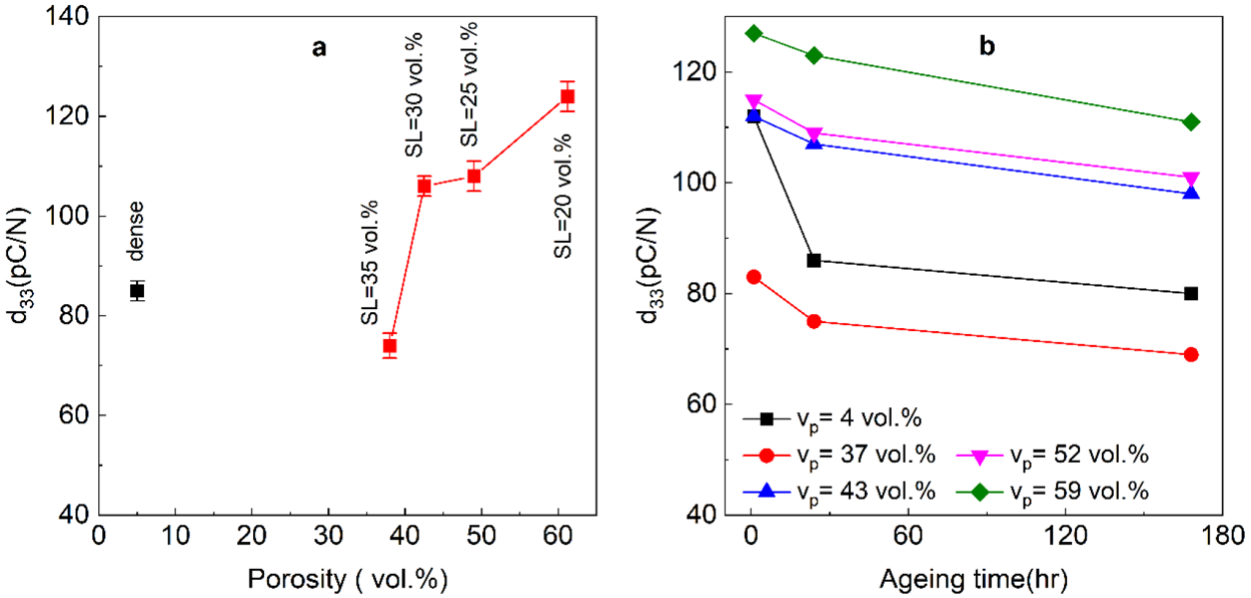
(a) Longitudinal piezoelectric charge coefficient, *d*_33_, as a function of porosity volume for the dense and freeze cast porous BFBT30 taken after 24 h. Error bars indicate the range of measured values from 3 to 4 samples. (b) Time-dependent reductions in *d*_33_ after poling for dense and porous BFBT30 ceramics.

The longitudinal piezoelectric coefficient, *d*_33_, of the dense and porous BFBT30 ceramics (3–4 samples of each group) with varying porosity measured 24 h after poling (poling direction as shown in Figure 1e) are shown in Figure 5a. All porous ceramics except for the *v*_p_ = 37 vol % sample had a greater *d*_33_ than that of dense BFBT30. The *d*_33_ of the dense BFBT30 was 85 ± 2 pC/N compared to 124 ± 3 pC/N for the 59 vol % porosity freeze cast sample. As the solid loading decreased, the alignment of the pores and ceramic channels in the microstructure to the freezing (and poling) direction improved (Figure S3), which has been shown previously to increase poling efficiency and could account for the increase in *d*_33_ with overall porosity volume in the freeze cast samples.^13^ Furthermore, highly aligned porosity within a ferroelectric ceramic matrix has been shown to reduce residual stress significantly, allowing for enhanced poling in BaTiO_3_ and increased extrinsic contributions to strain via a declamping effect.^14^ It should also be noted that the average grain size in porous specimens is larger than those of the dense specimens, which could also impact the piezoelectric properties, e.g., by altering grain clamping due to lower grain boundary densities; however, the large volume of porosity is likely to play a more significant role in this regard. Grain size, *t*, and domain size, *D*_*d*_, are also correlated such that  in dense ferroelectric ceramics, which can further affect the piezoelectric properties.^44^ However, there is currently little understanding of how large pore fractions may alter either the relationship between grain and domain size or piezoelectric properties. A finer domain structure was reported previously in porous compared to dense PZT;^45^ this was not correlated directly to the grain size although no significant change was reported with the introduction of porosity. Furthermore, improved *d*_33_ in porous samples may be linked to a lower fraction of Fe^3+^ ions being converted to Fe^2+^ ions relative to the dense BF-BT (Figure 4d) despite the porous sample that was investigated in the XPS study having a higher concentration of oxygen vacancies. Increased oxygen vacancies in BF-BT ceramics were also found to increase the *d*_33_ by enhancing the intrinsic and extrinsic contributions to piezoelectric properties.^46^

The *d*_33_ values of dense and porous BFBT30 samples, measured as a function of time after poling, are shown in Figure 5b. The initial measurements were carried out 4 h after poling, and all porous materials, except for the *v*_p_ = 37 vol % samples, exhibited a higher *d*_33_ compared to their dense counterparts. The highest value of *d*_33_ was 127 ± 2 pC/N for a porous specimen that had a solid loading of 20 vol % and porosity of 59 vol %, which dropped to 124 ± 3 pC/N after 24 h and 111 ± 2 pC/N after 1 week. The dense BF-BT showed a more rapid decline in *d*_33_, which dropped by around 20% from 112 ± 3 to 85 ± 2 pC/N after 24 h compared to the porous specimens that exhibited a decrease of less than 10% over the first 24 h. Over a period of 1 week (168 h), the *d*_33_ of dense ceramics dropped even further, reaching 80 pC/N, but the *d*_33_ of porous samples dropped only marginally, remaining at >110 pC/N for the *v*_p_ = 59 vol % sample.

**Figure 6:**
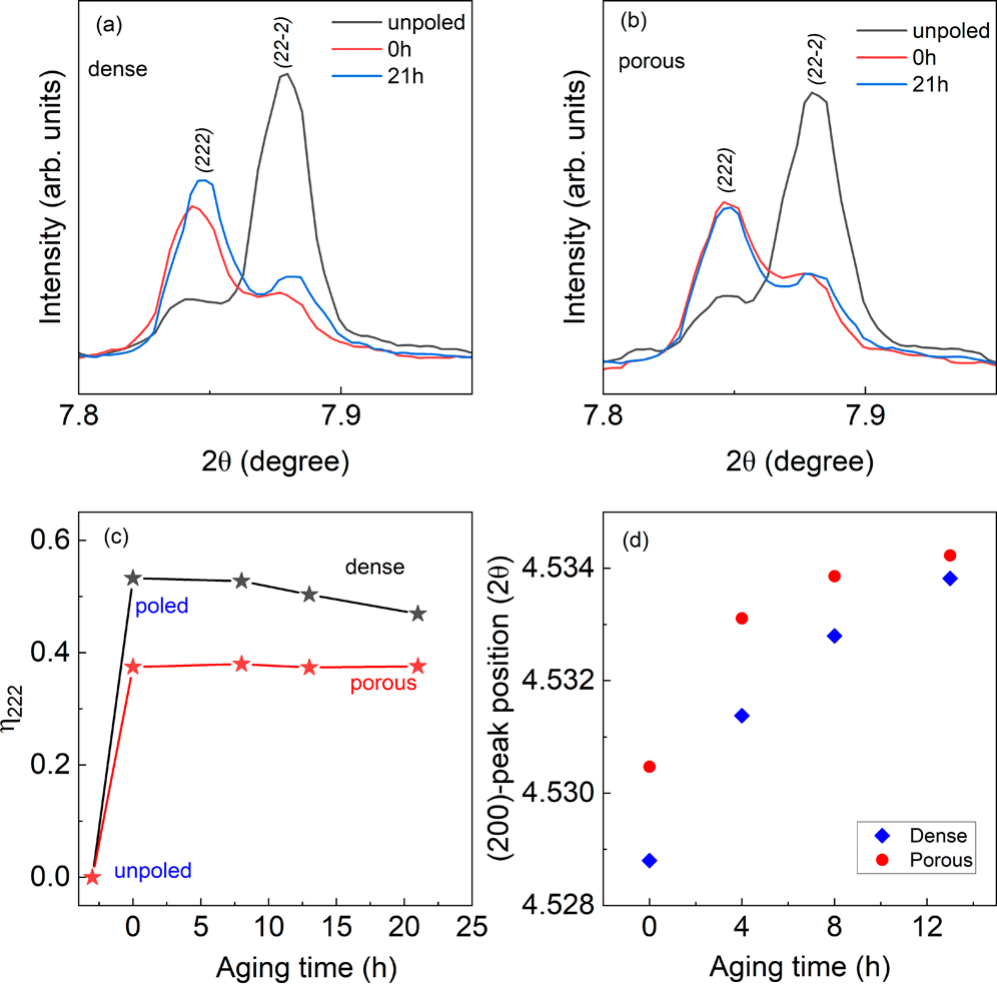
Selected (222) XRD peak profiles measured: (a) dense (*v*_p_ = 4 vol %) and (b) porous (*v*_p_ = 43 vol %) BF-BT ceramics at an azimuthal angle of ψ = 0° in the unpoled state, immediately after poling and after 21 h. (c) Fraction of domains switched in the poling direction calculated from the (222) peaks using eq 11 for dense and porous BFBT30, and (d) 200 peak positions of the poled dense and porous samples as a function of time after poling.

Synchrotron XRD experiments were used to investigate back-switching and lattice strain within dense and porous BF-BT as a function of time after poling to inform on the mechanism behind the observed aging effects. An XRD pattern at zero field was first taken of a dense (*v*_p_ = 4 vol %) and a porous (*v*_p_ = 43 vol %) sample before poling, and then subsequently up to 21 h after poling. Pseudo cubic (222) reflections at an azimuthal angle ψ = 0° (i.e., in the direction of poling) are shown in Figure 6a and b for dense and porous BF-BT, respectively; the (111) and (200) peaks are shown in Figure S6. The (222) peaks are chosen given that the presence of 71° and 109° ferroelectric domains in the rhombohedral structure and external electric field-driven domain switching can be observed in change in relative intensity of 222/222 peak intensities.^47^ The fraction of domains switched in the poling direction (η_222_) was calculated from the (222) peak intensities using^47^
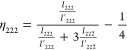
11where *I*_*hkl*_ and *I*_*hkl*_^*’*^ are integral intensity of the (hkl) diffraction peaks of the poled and unpoled sample, respectively. Prior to poling, the domains were randomly oriented, which resulted in a switched fraction calculated using eq 11 of zero for both the dense and porous material. Both samples were then poled at 5 kV/mm electric field for 5 min and XRD data were collected as a function of time after poling. Both dense and porous exhibit significant fractions of domain reorientation upon poling; see Figure 6c. The higher poling fraction for the dense compared to the porous BF-BT should lead to a higher *d*_33_, which was not observed in the room temperature measurements (Figure 5). This may be due to different poling conditions and sample-to-sample variability as well as local field inhomogeneities in the porous materials. After 21 h postpoling, the porous sample showed minor domain relaxation, whereas the dense sample showed roughly 11% domain back-switching compared to immediately after poling (Figure 6c). It was also observed that as well as domain reorientation there was a shift in peak position toward lower 2θ angle; see Figure 6d for the 200-peaks, indicating the development of lattice strain in that crystallographic direction when poled. The 200-lattice strain for the dense was higher than that for the porous BF-BT when poled under the same field, but relaxed to a similar position after 21 h. The rate of relaxation was therefore faster for the dense material. A recent study found that aging occurred faster in BF-BT ceramics with a higher concentration of oxygen vacancies.^46^ In this work, the oxygen vacancy concentration increased for the porous BF-BT due to the enhanced bismuth loss during sintering (Figure 4), however, the aging rate decreased with porosity (Figure 5b). This suggests that the reduction in aging rate with porosity is likely due to microstructural effects such as pores reducing residual stress that leads to domain back-switching after poling rather than a mechanism related to the local defect structure.

**Figure 7:**
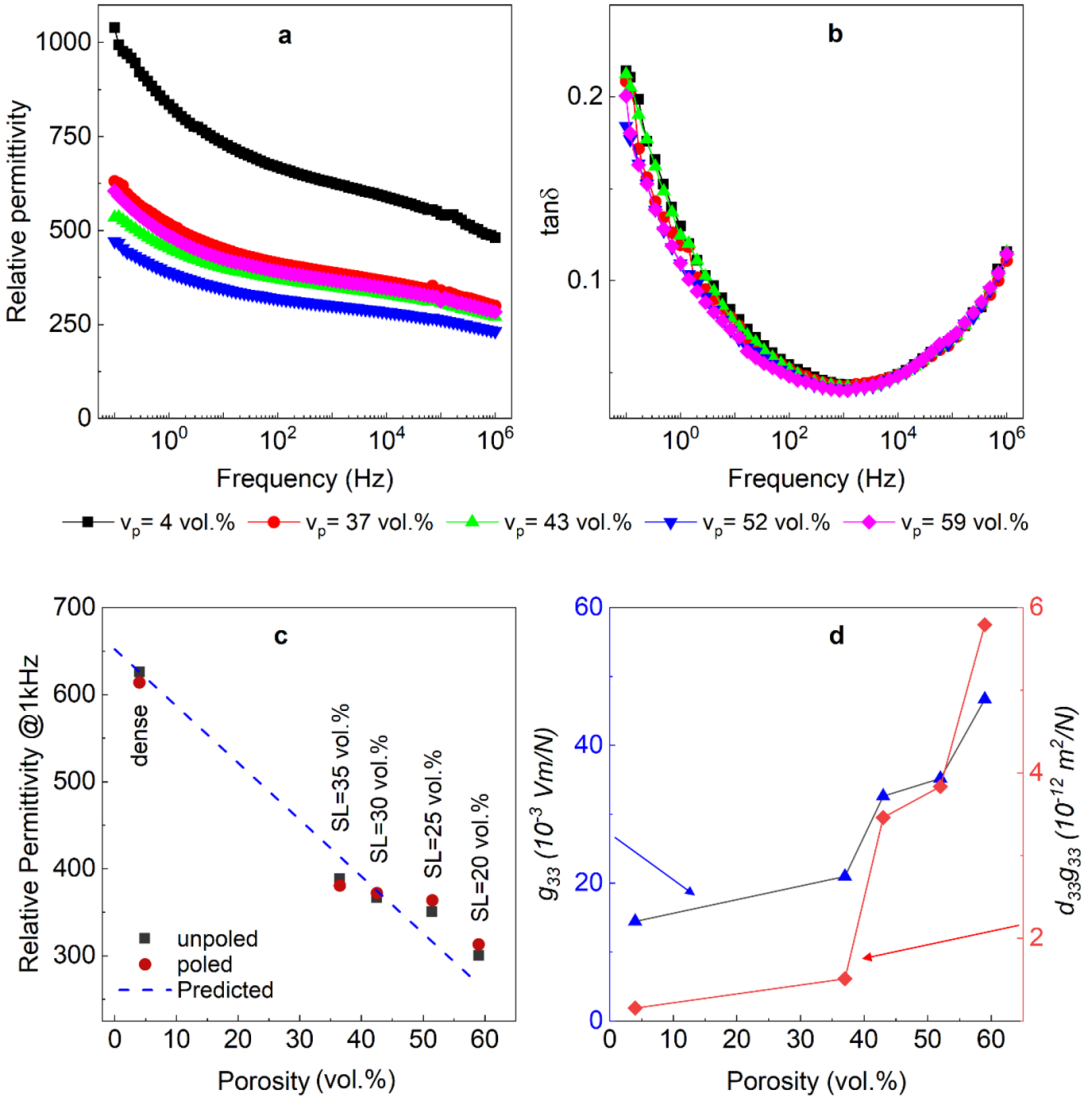
(a) Relative permittivity and (b) dielectric loss as a function of frequency for the dense and porous BFBT30 ceramics, (c) relative permittivity (at 1 kHz) as a function of porosity for the unpoled (black block), poled (red circle), and predicted value using eq 12 (dashed blue line) of dense and porous samples, and (d) *g*_33_ and *d*_33_*g*_33_ values as a function of porosity.

The frequency dependent relative permittivity and dielectric loss (tan δ) of dense and porous BFBT30 at room temperature are shown in Figure 7a and Figure 7b, respectively. For all samples, the relative permittivity was higher at lower frequencies and decreased with increasing frequency. For the entire frequency range, all of the porous specimens had lower relative permittivity than the dense BFBT30 ceramics, with permittivity decreasing as a function of porosity in all cases. Dielectric loss was observed to be lowest around 1 kHz and increased for frequencies above 3 kHz, which may be due to dipolar losses associated with re-entrant relaxor ferroelectric behavior observed recently in BF-BT.^48^ At frequencies below 500 Hz the loss tangent is inversely proportional to frequency and is attributed to the effects of DC conductivity, which also leads to an increase in the measured permittivity in this region. The dielectric loss was not significantly affected by the presence of a porosity. The AC conductivity is shown in Figure S7, which shows a slight decrease in conductivity with porosity.

Figure 7c shows the effect of the porosity on the relative permittivity of poled and unpoled BFBT30 ceramics at 1 kHz. Dense ceramics had relative permittivity, ε_33_^*T*^/ε_0_ = 626 at 1 kHz, which decreased to around ε_33_^*T*^/ε_0_ = 300 for the *v*_p_ = 52% (SL = 20 vol %) sample. The permittivity for the highest porosity sample is slightly higher than that predicted using a linear rule-of-mixtures for a parallel connected composite microstructure:
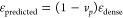
12which gives a predicted relative permittivity (poled with a blue dotted line in Figure 7c) of ε_33_^*T*^/ε_0_ = 267 using the input value of *ε*_*dense*_ = 652 (the extrapolated value at 100% theoretical density from the measured dense sample with a theoretical density of 96%). The agreement between the experimental data and eq 12 indicates that the freeze cast material was well aligned in the direction of measurement. However, the actual microstructures of the freeze cast materials are not perfectly aligned and contain pores within the ceramic lamellae (see Figure S3), which can have a relatively large impact toward reducing the measured permittivity. As such, even for a well-aligned porous material the permittivity typically falls someway below that predicted by eq 12.^49^ One possible explanation is that the assumption of permittivity for the BF-BT phase being independent of the volume of porosity in this model is incorrect and that the actual average permittivity of the BF-BT increases when porosity is introduced.

Another interesting observation is that the relative permittivity of the poled specimen was lower than that of the unpoled dense (*v*_p_ = 4 vol %) and *v*_p_ = 37 vol % freeze cast specimens, whereas it increased for the other freeze cast specimens (*v*_p_ = 43 vol %, 52 vol %, and 59 vol %), see Figure 7c. This may be related to the anisotropic dielectric properties of the material and domain dynamics during and after poling. Anisotropic properties can account for a reduction in the permittivity with poling in ferroelectrics with a rhombohedral symmetry,^50^ whereas poling can increase domain size and therefore reduce the contribution to permittivity from domain walls, thereby decreasing the overall permittivity.^51^

The microstructural and piezoelectric properties are summarized in Table 1, including the longitudinal piezoelectric coefficient *d*_33_, relative permittivity ε_33_^*T*^/ε_0_ at 1 kHz, longitudinal voltage coefficient *g*_33_, and energy harvesting figure of merit, *d*_33_*g*_33_. The voltage sensitivity and harvesting figure of merit, plotted as a function of porosity in Figure 7d, increased with increasing porosity by factors of three and five times, respectively, compared to the dense material due to the combined effects of the reduction in ε_33_^*T*^ and increase in *d*_33_.

**Table 1:** Microstructural Properties (Porosity and Mean Lamellar Thickness of Porous Sample) and Small Signal Coefficient (Longitudinal Piezoelectric Coefficient *d*_33_, Maximum Permittivity at 1 kHz at Room Temperature, Piezoelectric Voltage Coefficient *g*_33_, and Energy-Harvesting Figure of Merit, *d*_33_*g*_33_) for Dense and Porous Samples

solid loading (vol %)	porosity (vol %)	lamellae thickness (μm)	*d*_33_ (pC/N)	ε_33_^*T*^/ε_0_ at 1 kHz	*g*_33_ = *d*_33_/ε_33_^*T*^ (×10^–3^ V m/N)	*d*_33_*g*_33_ (×10^–12^ m^2^/N)
dense	4		85	626	14.6	0.95
35	37	37.9	75	388	21.5	1.6
30	43	18.3	107	367	32.6	3.5
25	52	10.1	109	350	35.4	3.8
20	59	7.67	124	300	46.4	5.7

### 3.3 Ferroelectric Properties

**Figure 8:**
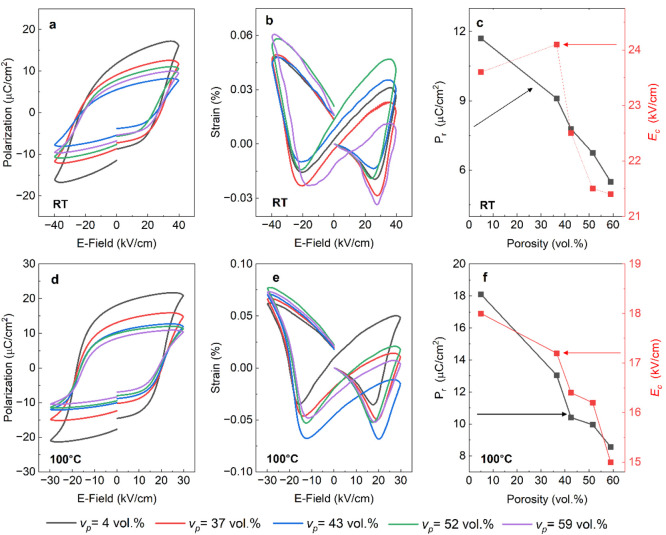
(a) Polarization–field (*P*–*E*) and (b) strain–field (*S*–*E*) hysteresis loops for dense and porous BF-BT measured up to 40 kV/cm and (c) remnant polarization (*P*_r_, black square) and coercive field (*E*_C_, red circle) as a function of porosity, all measured at room temperature (RT). (d) *P*–*E* and (e) *S*–*E* loops up to 30 kV/cm measured at 100 °C and (f) extracted *P*_r_ (black square) and *E*_C_ (red circle) values as a function of porosity at 100 °C.

The *P*–*E* and *S*–*E* hysteresis loops for dense and different porous samples measured at room temperature (RT) are shown in Figure 8a and Figure 8b, respectively. The dense pellet survived electric fields greater than 60 kV/cm, whereas dielectric breakdown occurred in the porous samples above 40 kV/cm. While the loops are not fully saturated, the data are plotted at the lower field to enable comparison between the dense and porous materials. The remnant polarization and coercive field with pore fraction at room temperature shown in Figure 8c obtained from these loops are therefore only indicative of the trend. In the Supporting Information, the *P*–*E* and *S*–*E* loop measurements for the dense (Figure S8) and porous BF-BT (Figure S9) are presented to illustrate the effects of varying porosity over a wide temperature range (22 to 175 °C) at the 30 kV/cm field amplitude. Figure 8d and Figure 8e show the *P*–*E* and *S*–*E* hysteresis loops at 100 °C for dense and freeze cast BF-BT samples. Dielectric breakdown occurred in the sample with *v*_p_ = 59 vol % above 30 kV/cm at 100 °C and so for comparison loops are only plotted to a peak field of 30 kV/cm. Even when the field strength was reduced to 30 kV/cm, the *P*_s_ and *P*_r_ values measured at 100 °C were higher for both dense and porous materials when compared to the room temperature values. When measured at 100 °C, bipolar electrostrain values slightly increased when compared to RT values.

The coercive field gradually decreased with an increasing porosity (Figure 8c,f). This could imply that adding porosity to the matrix facilitates domain mobility and improves the ferroelectric response. This correlates with reduced residual stress that enables increased domain wall motion,^14^ without compromising the measured field-assisted long-range FE order at room temperature and at elevated temperatures. It is also consistent with the observed increase in *d*_33_ of the porous compared to the dense BF-BT; see Figure 5. The absolute value of the coercive field decreased with increasing temperature for all samples, leading to enhanced electric field induced ferroelectric switching with a slim hysteresis loop. A recent study on a similar composition showed that the rise in polarization with temperature may be attributable to an abnormal increase in rhombohedral distortion, which is associated with the re-entrant relaxor ferroelectric transition when heated from room temperature to ∼200 °C.^48^

The saturation (*P*_s_) and remnant polarization (*P*_r_) values of the porous ceramics were lower than those of dense materials at both room (RT) and elevated temperatures due to the lower volume of active ferroelectric material and the high volume of low permittivity pores. At RT and 40 kV/cm field strength, the *P*_*s*_ and electrostrain of the dense specimen was 16 μC/cm^2^ and 0.05% respectively (Figure 8a). As the porosity increased, *P*_s_, decreased to 11.8 μC/cm^2^ and 7.6 μC/cm^2^ for the *v*_p_ = 37 vol % and *v*_p_ = 43 vol % samples, respectively, before a slight increase to ∼9.5 μC/cm^2^ when *v*_p_ > 50 vol %. Despite having higher porosity, the *v*_p_ = 52 vol % and *v*_p_ = 59 vol % porous samples had slightly higher polarization (*P*_s_ and *P*_r_) and electrostrain values at both RT and 100 °C (*S*_pos_ = 0.06%, *S*_neg_ = −0.02%, *S*_total_ = 0.08% at RT and *S*_pos_ = 0.08%, *S*_neg_ = −0.05%, *S*_total_ = 0.13% at 100 °C) than the *v*_p_ = 43 vol % sample; see Figure 8b,e, which we attribute to the highly aligned microstructure and improved polarizability of the active material. The electrostrain values of the porous samples with *v*_p_ = 52 vol % and *v*_p_ = 59 vol % were also higher than the electrostrain of dense samples: *S*_pos_ = 0.05%, *S*_neg_ = −0.02%, *S*_total_ = 0.06% at room temperature and *S*_pos_ = 0.06%, *S*_neg_ = −0.03%, *S*_total_ = 0.09% at 100 °C. Improved electrostrain in the porous samples may be associated with higher concentration of oxygen vacancies. An experimental and simulation study on BNT-based piezoceramics demonstrated that oxygen vacancies, in conjunction with an external electric field, induced strong atomic hybridization, large ionic displacement, in-plane rotation of oxygen octahedra, and energy level shifts, which enhanced the electrostrain by ∼2.3% at elevated temperatures of 220 °C.^52^

**Figure 9:**
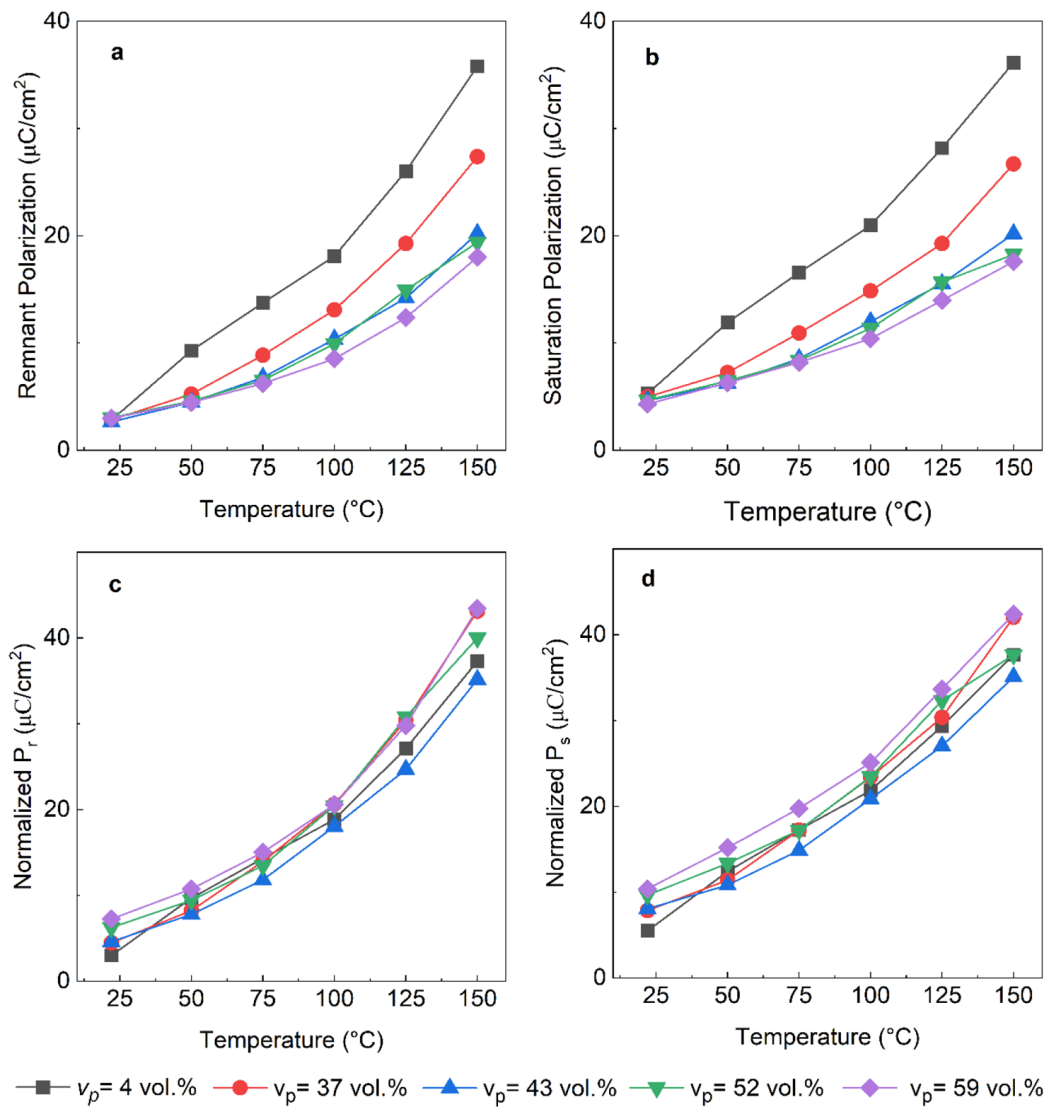
Temperature dependent (a) remnant polarization (*P*_r_) and (b) saturation polarization (*P*_s_) for dense and porous BFBT30 respectively. (c) *P*_r_ and d) *P*_s_ values normalized relative to the volume of active material, i.e., by dividing the measured polarization by the relative density of the sample as a function of temperature.

The temperature dependence of remnant and saturation polarization values for dense and porous BFBT30 ceramics is shown in Figure 9a and 9b, respectively. Both *P*_r_ and *P*_s_ increased with the temperature for all samples. The normalized values of *P*_s_ and *P*_r_ as a function of temperature are shown in Figure 9c and Figure 9d, which were calculated by dividing the measured polarization values by the relative density of the sample to account for the decrease in active ferroelectric ceramic material in the porous samples. Similar to the effect of porosity on the permittivity, it was anticipated that for a well aligned porous microstructure the normalized polarization values should be close to but not exceed those of a dense sample of the same composition.^49^ However, here the normalized remnant and saturation polarization values of the porous BFBT30 samples slightly increased compared to those of the dense reference suggesting that the high volumes of porosity could be facilitating easier polarization switching leading due to relaxed elastic constraints. This increase in the normalized polarization cannot be explained through an increase in conductivity, which decreased slightly with porosity (Figure S7). A similar approach was used by Khansur et al.^32^ to normalize measured polarization values with respect to the change in electrode area due to the presence of porosity in PZT and no significant change was observed for the PZT ceramics with unaligned, uniformly distributed porosity.

**Figure 10:**
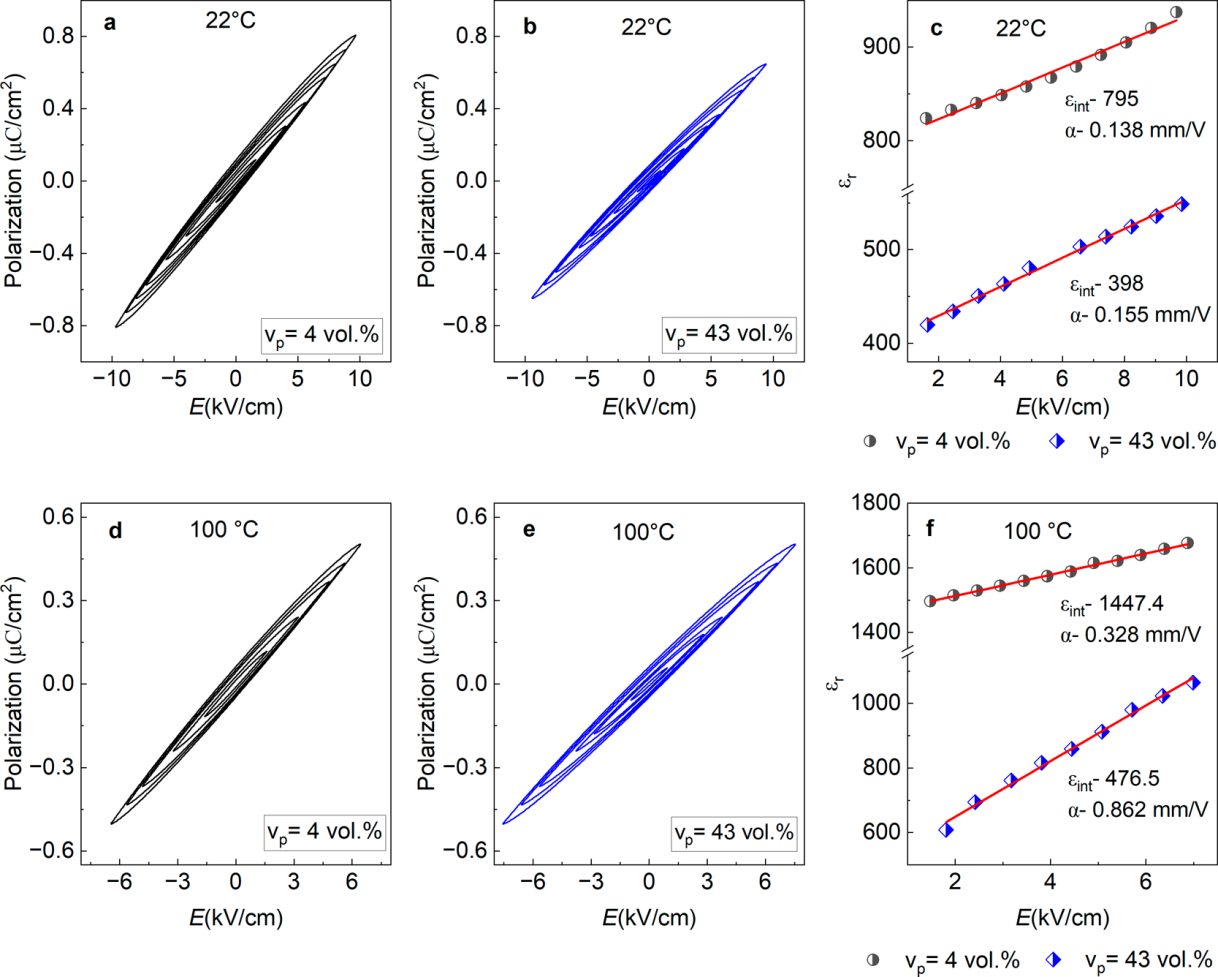
Polarization vs electric field loops in the subcoercive field region (1–10 kV/cm) at (a, b) 22 °C and (d, e) 100 °C for *v*_p_ = 4 vol % (dense) and *v*_p_ = 43 vol %. Rayleigh coefficients were calculated in eq 6 for the dense BF-BT and porous *v*_p_ = 43 vol % at (c) 22 °C and (f) 100 °C, respectively.

To further evaluate the effect of porosity on polarization switching facilitated by easier domain wall motion, Rayleigh analysis was performed on both dense (*v*_p_ = 4 vol %) and porous (*v*_p_ = 43 vol %) samples at RT and 100 °C. The lattice contribution is reversible whereas domain wall contribution has both reversible and irreversible components.^53^ The extrinsic contribution increases with increasing field amplitude and plays an important role in the piezoelectric and dielectric properties. Figure 10 shows the low field *P*–*E* hysteresis loops for these BF-BT ceramics at room temperature of 22 °C (Figure 10a,b) and at 100 °C (Figure 10d,e) measured at subcoercive electric field amplitudes in the range 1.5–10 kV/cm at 1 Hz frequency. The intrinsic *ε*_int_ (initial permittivity) and extrinsic α_ε_ (Rayleigh) coefficients were calculated by using eq 6 from the data shown in Figure 10c for RT and Figure 10f at 100 °C. A higher value of the Rayleigh coefficient α_ε_ was observed for the *v*_p_ = 43 vol % porous sample (0.155 mm/V) compared to that of the dense specimen (0.138 mm/V) whereas the zero-field relative permittivity ε_int_ = 398 for the porous sample was less than that of the dense sample (ε_int_ = 795) at room temperature, see Figure 10c. The value of the α_ε_ coefficient for the porous sample increased sharply to α_ε_ = 0.862 mm/V, 2.5 times larger than that of the dense ceramic (*α*_*ε*_ = 0.328 mm/V) as temperature increased to 100 °C, which is close to the Rayleigh coefficient reported for the BaTiO_3_ modified BiFeO_3_–PbTiO_3_ piezoceramic system (1.1 mm/V).^54^ Increases in the rhombohedral distortion with temperature may play a key role in the large increase in *α*_*ε*_ coefficient at 100 °C by making it possible to convert the ferroelectric domain structure to a relaxor state.^48^ Another possible contributor is the 30% drop in the coercive field for the porous material at this temperature compared to a 23% drop for the dense sample.

## 4 Discussion

### 4.1 Role of the Porosity in Dielectric, Piezoelectric, and Ferroelectric Properties

The addition of aligned porosity to BFBT30 ceramics reduced the relative permittivity at 1 kHz from ε_33_^*T*^/ε_0_ = 626 for the dense to ε_33_^*T*^/ε_0_ = 300, whereas the corresponding *d*_33_ value increased from 85 to 124 pC/N for the 59 vol % porosity sample. This resulted in a significant increase in piezoelectric voltage sensitivity, *g*_33_ = 46.5 × 10^–3^ Vm/N, and energy harvesting figure of merit *d*_33_*g*_33_ = 5.7 × 10^–12^ m^2^/N, compared to the dense BF-BT, where *g*_33_ = 14.6 × 10^–3^ Vm/N and *d*_33_*g*_33_ 0.95 × 10^–12^ m^2^/N. The increase in sensing and harvesting figures of merit are larger than expected due to the unusual increase in *d*_33_ with porosity coupled with the decrease of ε_33_^*T*^ with the introduction of aligned porosity in BF-BT. Comparable *d*_33_ values to the dense system have been reported previously for highly aligned, porous freeze cast piezoceramics^11,34,45^ due to the promotion of a high poling efficiency not achievable in unaligned porous piezoceramics,^55^ but it is unusual for the piezoelectric properties to increase relative to a dense material.

The underlying mechanisms for the increase in *d*_33_ with porosity observed in this study can be interpreted through ferroelectric measurements. All the porous BF-BT samples studied here had lower absolute values of remnant and saturation polarization relative to dense samples measured at the same field amplitude, which was expected due to the lower volume of active ferroelectric material present. However, the hysteresis loops of the porous BFBT30 reached saturation at lower fields than those of the dense samples (Figure 8). Furthermore, in comparison with the dense reference material, the remnant and saturation polarization values, adjusted according to the volume fraction of the ferroelectric in the total volume of the sample of the porous BFBT30 samples, showed a slight increase relative to the dense material (Figure 9). This may be related to porous samples having a lower coercive field, for example, 21.4 kV/m for *v*_p_ = 59 vol % sample compared to 23.6 kV/cm for dense samples at RT, indicating that porosity enhances the domain mobility for the same given field. It should be noted that the *P*–*E* loops at RT were not fully saturated due to dielectric breakdown of the porous samples at the fields required for full saturation and the comparison here therefore assumes the data is indicative of the trends that would be observed for fully saturated loops. In barium titanate, porosity was found to significantly reduce residual stress and facilitate domain switching,^56^ which also played a role here. Improvements in the *d*_33_ and polarizability of the porous samples could also be attributed to the larger grain size in comparison to the dense ceramics, which has been shown in other material systems to relate to the size of domains.^57^ The only previous report of higher piezoelectric properties in a freeze cast porous system relative to the dense system was in PZT, attributed to a smaller domain size in the porous material after sintering.^45^ It is likely that the magnitude of the different contributions to the observed effects of porosity on the piezoelectric properties of ferroelectric material differs depending on the material system being studied.

### 4.2 Effect of Porosity on the Aging Effect on Piezoelectric Properties

Not only was the largest longitudinal piezoelectric charge coefficient, *d*_33_, observed in the most highly porous samples, but high volumes of porosity also reduced the effect of aging on the BFBT30 compared to the dense material (Figure 5). The origin of aging and potential mechanisms for porosity mitigating aging in these materials is now discussed.

#### 4.2.i Microstructural Factors That Contribute to the Aging Process in Ferroelectric Ceramics

Charged defects, volume effects, domain walls, or grain boundary effects can all contribute to a restabilization of the domain configuration that is responsible for the aging behavior^58,59^ Defects such as space charge, charged defects, dipolar defect associates, and so on, are very common in Bi-based piezoceramics, affecting their electrical properties. These defects form during the sintering process because of the evaporation of volatile components (Bi_2_O_3_) in the composition. The loss of bismuth oxide creates V_Bi_‴ and V_O_^••^ charged defects during sintering. The oxygen vacancies, V_O_^••^, mainly originate from bismuth loss and the conversion of Fe^3+^ into Fe^2+^.^60^ Presence of V_Bi_‴ and V_O_^••^ defects leads formation to defect dipoles that are aligned with the *P*_*s*_ direction and likely pinned to the domain wall boundaries. Oxygen vacancies are evident in both porous and dense samples, as shown in Figure 4. Nevertheless, despite the greater reduction of Fe^3+^ to Fe^2+^ in the dense, the greater number of oxygen vacancies in the porous samples indicate greater bismuth loss. Recent work^46^ demonstrated that higher oxygen vacancy concentrations increase the rate of aging of the piezoelectric properties of dense BF-BT. However, we found that aging effects were reduced in the porous BF-BT despite their higher oxygen vacancy concentration, indicating that the porous microstructure has a greater influence on these properties. The grain boundary effect, also known as the space charge effect, describes polarization aging caused by the accumulation of space charge (point defects) near grain boundaries.^61^ The migration and accumulation of charge point defects in dense and porous specimens may differ on and after poling, related to the difference in grain size and mechanical clamping at grain boundaries.

#### 4.2.ii Residual Stress Influencing the Depoling Rate

Residual stress has a significant effect on the functional properties of dense piezoceramics which is influenced both by the grain size and microstructural features such as porosity.^62^ A high residual stress after poling may induce the time-dependent relaxation (domain back-switching) of domains in the dense BFBT30 ceramic, leading to the decline in *d*_33_ after poling as observed here (Figure 5b and Figure 6) that appears to be released by the reduced elastic constraint in the porous materials. While residual stress was not directly measured here there are several indications that it is playing a role in the differing aging behavior of the dense and porous BFBT30 ceramics. First, the nonlinear domain wall contribution (extrinsic) was found to be higher for the porous BFBT30 compared to the dense material at both room and elevated temperatures, as shown by the Rayleigh analysis in Figure 10. For the extrinsic contribution to the piezoelectric properties, mechanical clamping plays a vital role in resisting the non-180° domain (ferroelastic) wall motion in thin films, single crystals and dense ceramics.^62−64^ Uniformly porous barium titanate ceramics modeled using the finite element method were shown to have a lower residual stress and local field distribution as a function of porosity that cause changes in the phase fraction of the tetragonal and orthorhombic ratio.^65^ Recently, it has also been reported that strongly orientated porous samples of freeze cast barium titanate have reduced residual intergranular stresses in comparison with dense materials, which results in a 2-fold increase in the extent of domain switching according to results obtained using in situ synchrotron diffraction.^56^ High volumes of aligned pores release the constraint of the surrounding polycrystalline matrix, resulting in greater domain switching response and a decrease of intergranular stresses in the highly porous freeze cast sample, which explains the faster saturation of the *P*–*E* loops observed in the porous sample compared to the dense BFBT30. Furthermore, in comparison with dense samples, the porous BFBT30 has larger average grain sizes, which is another reason to anticipate lower intergranular stresses, consequently enhancing the domain mobility.

## 5 Conclusions

In this paper, porous and dense 0.70BiFeO_3_ + 0.30 BaTiO_3_ + 1 mol % MnO_2_ (BFBT30) materials were fabricated and investigated for their piezoelectric and ferroelectric properties from room temperature to 175 °C. There was no appreciable difference in crystal structure according to XRD data of dense and porous samples. Freeze casting was used to create aligned porous materials, with improvements in alignment observed as the solid loading of freeze cast suspensions decreased and the overall final porosity increased. Immediately after poling, the *d*_33_ of porous and dense samples were comparable; however, the aging impact was significantly more pronounced in dense compared to the highest porosity samples. BF-BT with 59 vol % porosity had a significantly higher *d*_33_ of 124 pC/N measured 24 h after poling compared to 85 pC/N for the dense BF-BT, which reduced to 112 and 80 pC/N after 1 week for the porous and dense BF-BT, respectively. Ex situ high energy XRD patterns in transmission mode, collected as a function of time after poling, indicated that the aging effects were driven by back-switching of ferroelectric domains. The piezoelectric properties were also influenced by the differences in defect structures between dense and porous samples with a higher oxygen vacancy concentration observed in the porous BF-BT arising from bismuth loss during sintering and the reduction in Fe^3+^ valence. Contrary to other reports on oxygen vacancies in dense BF-BT, this did not increase the rate of aging in the porous materials investigated here, likely due to the significant difference in local mechanical clamping within highly porous microstructures.

The freeze cast BF-BT ceramics were demonstrated to have excellent potential for high-temperature sensing and energy harvesting applications. This arose from the decrease in the permittivity as porosity increased coupled with the increase in *d*_33_ in the porous BF-BT samples, leading to a large improvement in the piezoelectric voltage sensitivity (*g*_33_ = 46.5 × 10^–3^ V m/N) and energy harvesting figures of merit (*d*_33_*g*_33_ = 5.7 × 10^–12^ m^2^/N) in the 59 vol % porosity BF-BT compared to those of the dense BF-BT (*g*_33_ = 14.6 × 10^–3^ V m/N and *d*_33_*g*_33_ = 0.95 × 10^–12^ m^2^/N). With the introduction of porosity, electric field dependent measurements showed a decrease in remnant and saturation polarization, *P*_r_ and *P*_s_, due to the lower volume of active ferroelectric material in the total bulk volume. Normalizing the polarization values to account for the pore volume, however, revealed that the porous BF-BT ceramic was slightly more polarizable than the dense material, which was consistent with data for the increased *d*_33_ and the effect of porosity on the permittivity of the samples. Electric field induced polarization and strain values increased with temperature for both porous and dense BF-BT. The porous material had a higher strain–field response (30% higher at 100 °C) than the dense, which is attributed to increased polarizability/extrinsic response (more domain motion) for a given volume, declamping effects, or a combination of the two. This study has demonstrated that the effect of porosity added to BF-BT yields additional effects to those typically observed in porous ferroelectric ceramics, and therefore this approach shows great promise for engineering improved materials for high temperature transducer applications.

## Supporting Information Available

Additional experimental data including X-ray diffraction patterns, scanning electron micrographs, X-ray photoelectron spectroscopy scans, sample conductivity, and polarization- and strain-electric field behavior (PDF)
