# Supplementary material for: Temperature-Dependent Ferroelectric Properties and Aging Behavior of Freeze-Cast Bismuth Ferrite–Barium Titanate Ceramics
Source: ACS Appl Mater Interfaces. 2024 Apr 5;16(15):19283–97. doi: 10.1021/acsami.4c03002 (PMC11040575; doi:10.1021/acsami.4c03002)
Supplement: Supplementary file 1 — am4c03002_si_001.pdf [file am4c03002_si_001.pdf]

Supporting information

Temperature-dependent ferroelectric properties and ageing behaviour of freeze-cast bismuth ferrite-barium titanate ceramics

Bastola Narayan[1*], Zihe Li[1], Bing Wang[2], Astri Bjørnetun Haugen[3], David Hall[2], Hamideh Khanbareh[1], James Roscow[1*]

[1]Department of Mechanical Engineering, University of Bath, Bath, UK

[2]Department of Materials, University of Manchester, Manchester, UK

[3]Department of Energy Conversion and Storage, Technical University of Denmark, Copenhagen, Denmark

* Corresponding Authors emails: nb958@bath.ac.uk, jir24@bath.ac.uk

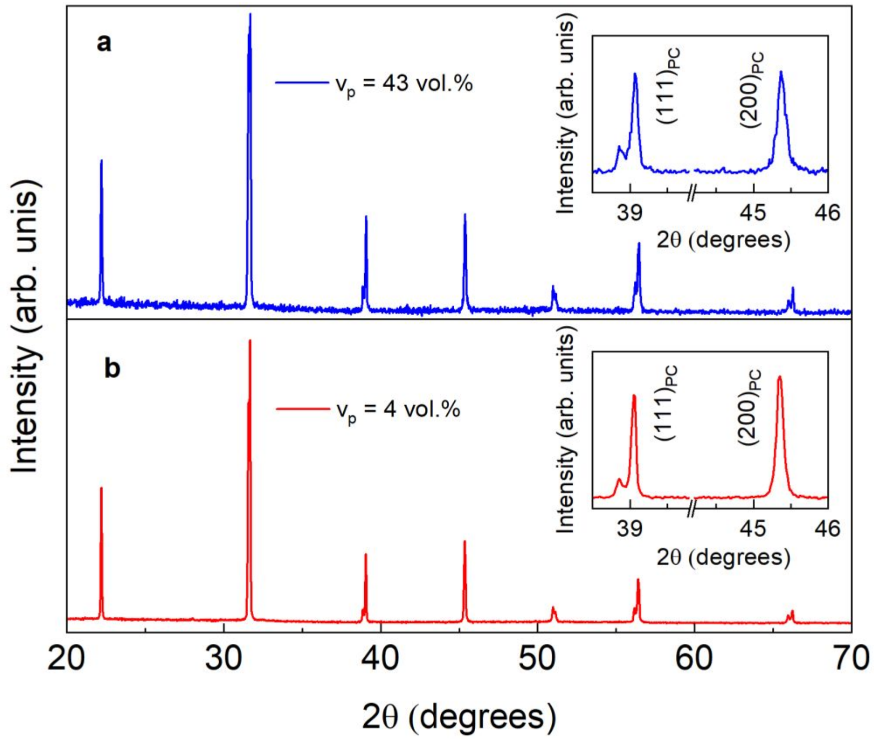

Figure S1: X-ray diffraction (XRD) pattern of a) porous ($v_p =$ 43 vol.%) and b) dense ($v_p =$ 4 vol.% porous unpoled powder BFBT sample (inset shows the pseudo-cubic (111) and (200) peaks of the same pattern). No significant change in structure was observed between the dense and the porous BFBT samples.

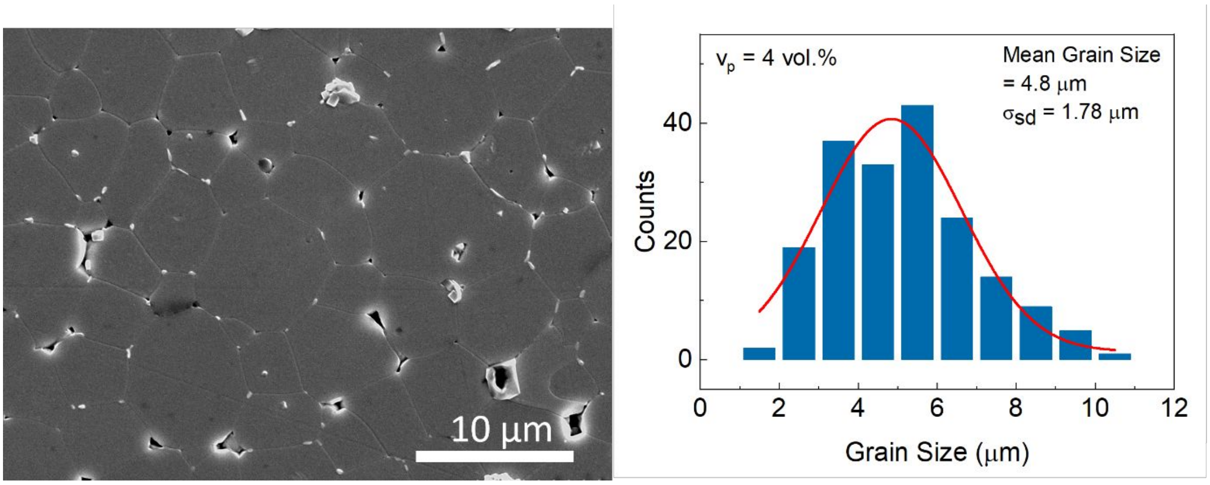

Figure S2: SEM micrograph of the polished surface of the dense specimen and the Gaussian fitting of the grain size (diameter) analysis conducted using ImageJ software.

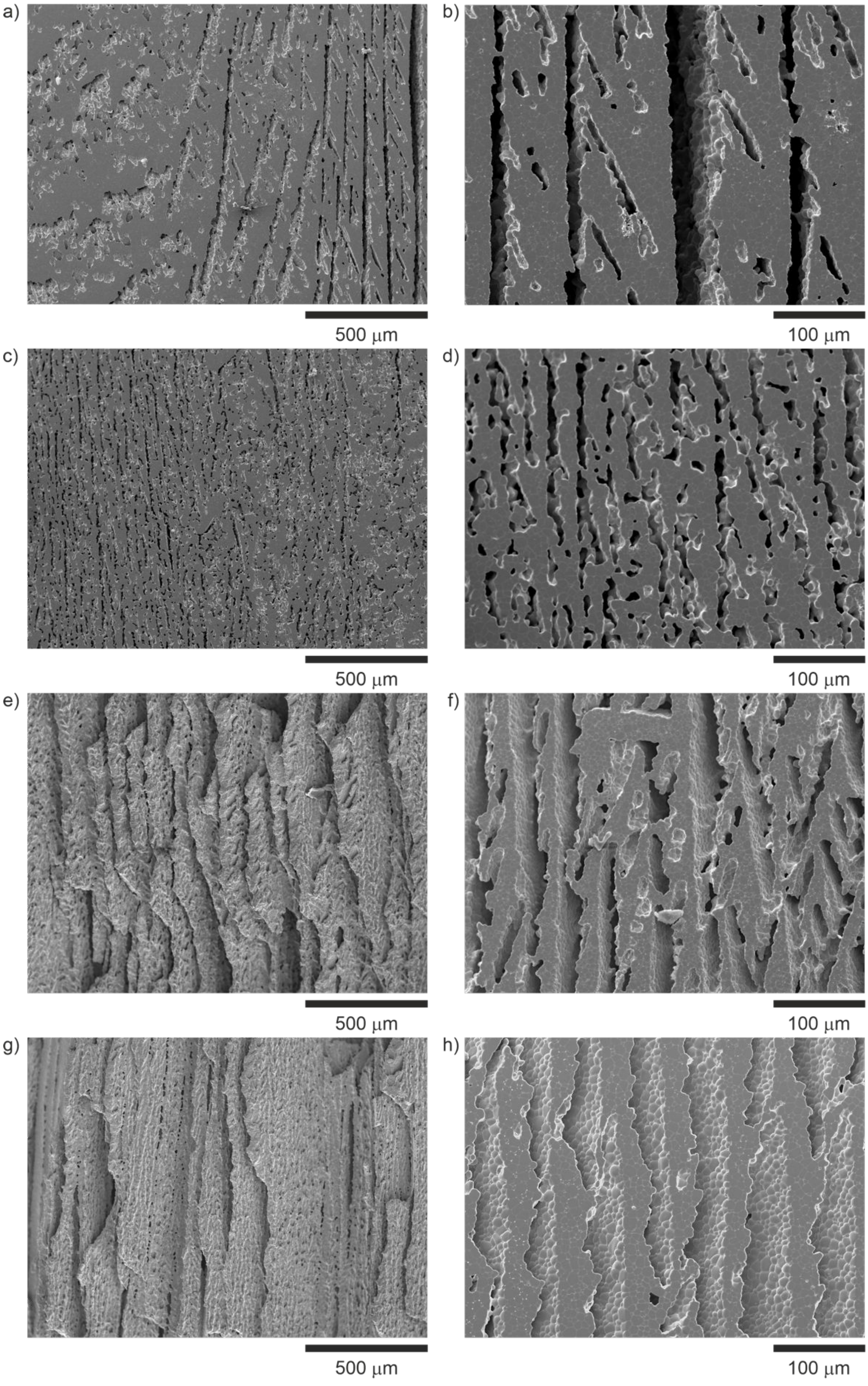

Figure S3: Scanning electron micrographs at different magnifications of freeze cast BFBT demonstrating the excellent microstructural alignment, particularly for the high porosity samples. a), b) solid loading, SL = 35 vol.% and porosity, v_p = 37 vol.%; c), d) SL = 30 vol.% and v_p = 43 vol.%; e), f) SL = 25 vol.% and v_p = 52 vol.%; and h), g) SL = 20 vol.% and v_p = 59 vol.%.

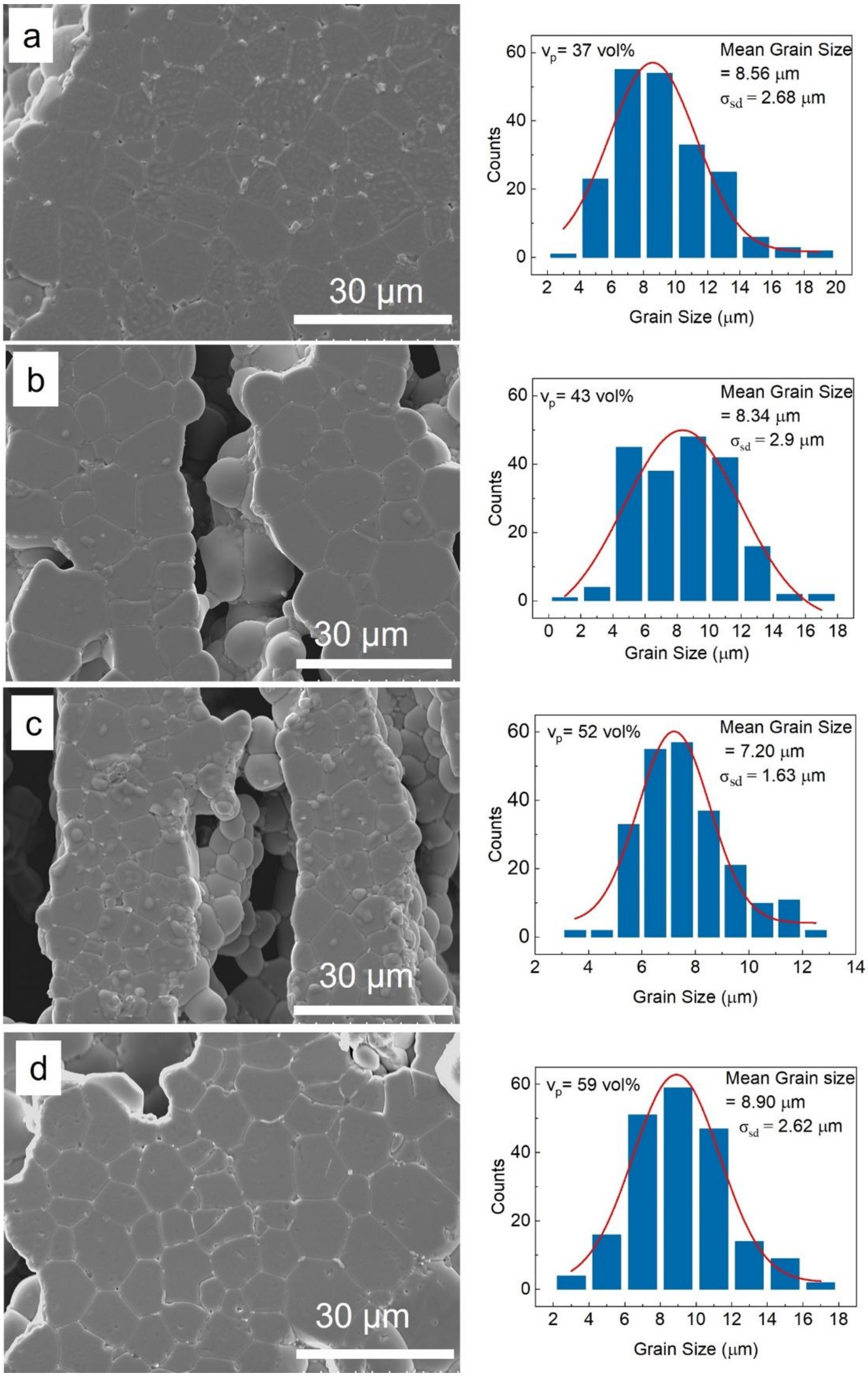

Figure S4: SEM micrographs of polished porous BFBT30 for grain size analysis with a) pore volume v_p = 37 vol.%, b) v_p = 43 vol. %, c) v_p = 52 vol.%, and d) v_p = 59 vol.%. The histograms on the righthand side of each SEM micrograph show the size distribution and Gaussian fitting of the grain size analysis using ImageJ software.

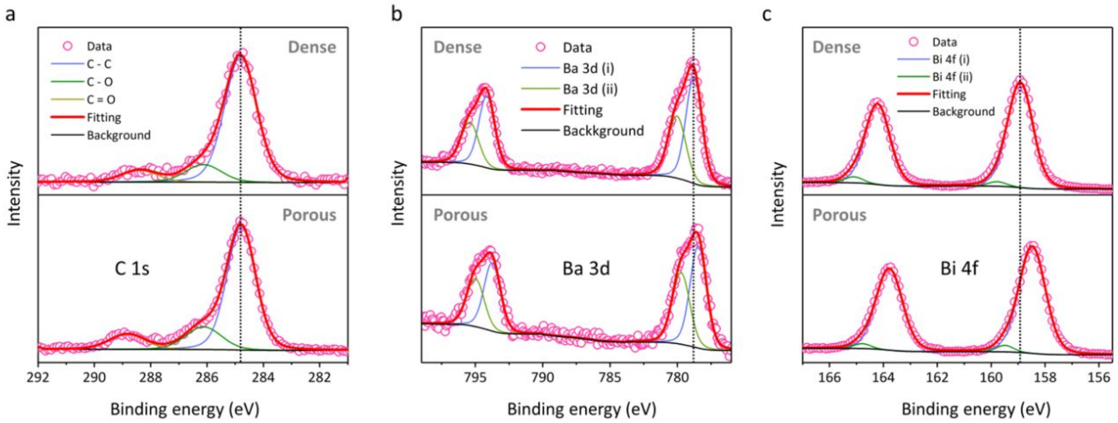

Figure S5: X-ray photoelectron spectra (XPS) of dense and porous (pore volume, v_p = 43 vol.%) BFBT30 ceramics: narrow scans of a) C 1s, b) Ba 3d and c) Bi 4f.

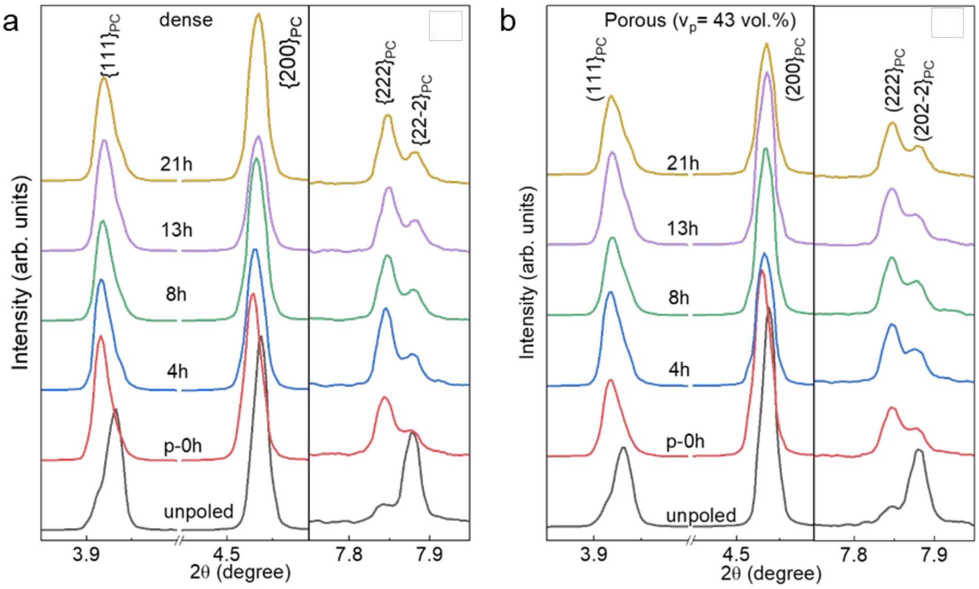

Figure S6: Pseudo-cubic (111), (200) and (222) XRD peaks taken in transmission mode using a synchrotron source before contact poling (unpoled) and at various times up to 21 hours after poling for a) dense BF-BT and b) porous (v_p = 43 vol.%) BF-BT. See the methods section in the main body of the manuscript for more detail.

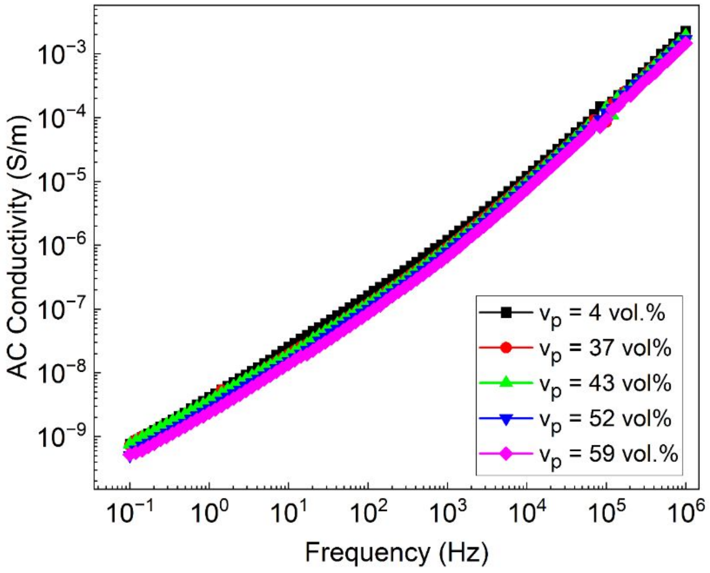

Figure S7: Frequency dependent AC conductivity of dense and porous BFBT30, measured with impedance spectroscopy. No significant change in the conductivity was observed between samples, with the small decrease with increasing porosity related to the insulating nature of the air in the pores.

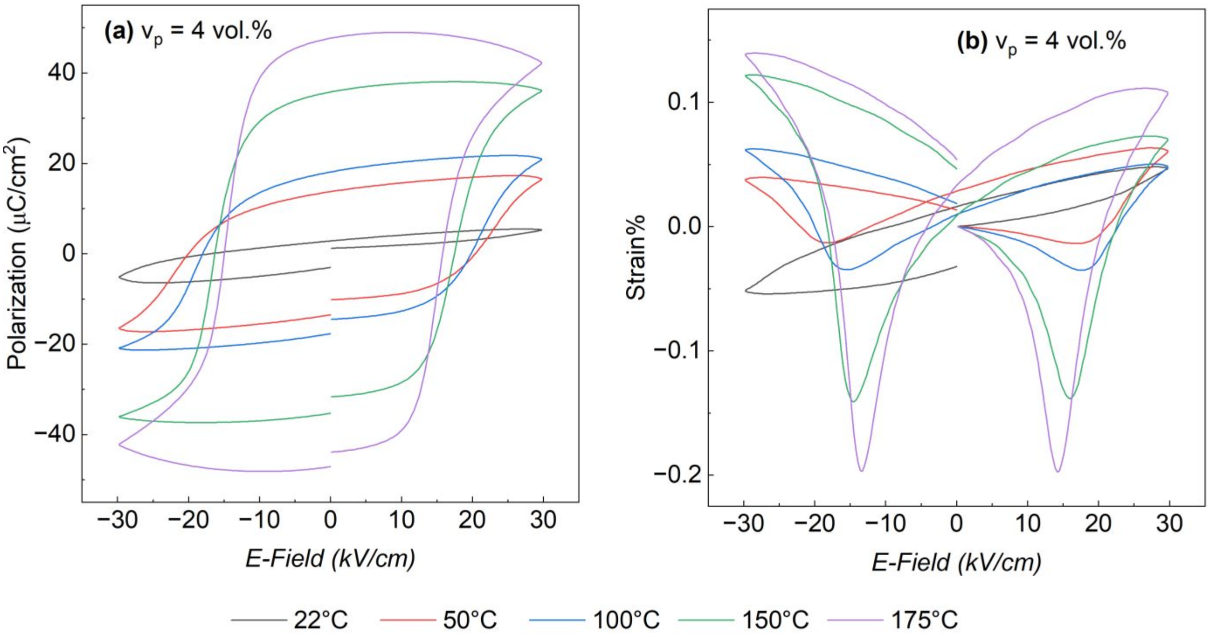

Figure S8: Temperature dependent a) polarization and b) bipolar strain hysteresis loops at 30 kV/cm field amplitude for the dense ($\rho_{rel} = 0.96$) BFBT30.

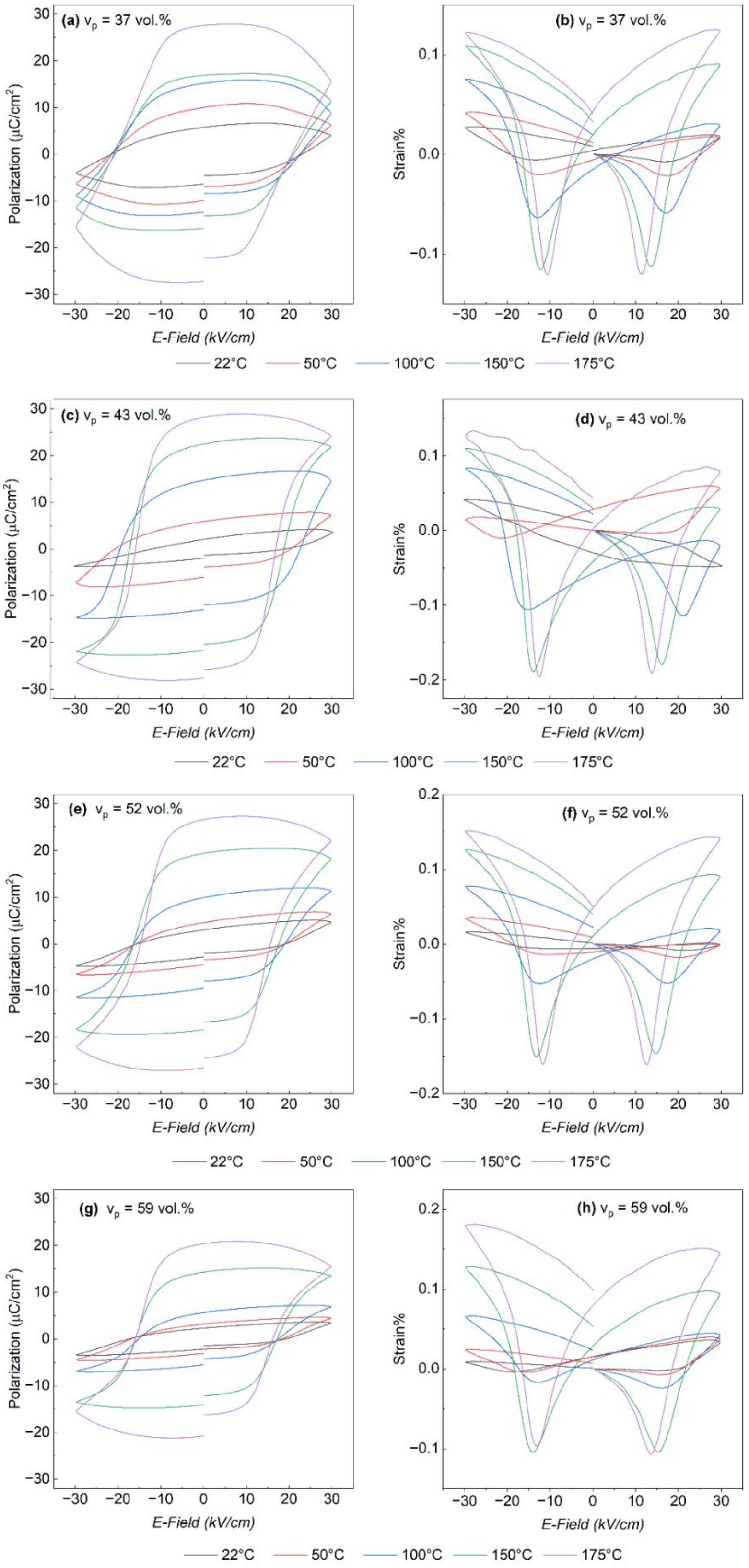

Figure S9: Temperature dependent polarization and bipolar strain hysteresis loops at 30 kV/cm field amplitude for freeze cast porous BFBT ceramics with a), b) v_p = 37 vol.%; c), d) v_p = 43 vol.%; e), f) v_p = 52 vol.%; and g), h) v_p = 59 vol.%. Polarization decreased with porosity as the volume fraction of ferroelectric material decreased but electro-strain increased due to the highly aligned nature of the microstructure in the poling direction and porosity-facilitated polarization switching.
